# The bacteriophage transcription factor Gp53 suppresses cell division and regulates *Pseudomonas aeruginosa* physiology

**DOI:** 10.1038/s42003-026-11031-z

**Published:** 2026-10-08

**Authors:** Ying Li, Xiaomin Li, Gukui Chen, Lixin Yuan, Hailong Zhong, Min Hui, Yongfeng Sun, Yufei Han, Shuqin Zhou, Shiwei Wang, Weina Kong

**Affiliations:** 1 Key Laboratory of Resources Biology and Biotechnology in Western China, Ministry of Education, Shaanxi Provincial Key Laboratory of Biotechnology, College of Life Sciences, Northwest University, Xi’an, China; 2 Department of Anesthesiology of The Second Affiliated Hospital, School of Medicine, The Chinese University of Hong Kong, Shenzhen & Longgang District People’s Hospital of Shenzhen, Shenzhen, China

**Keywords:** Bacteriophages, Molecular biology

## Abstract

*Pseudomonas aeruginosa* is a highly drug-resistant pathogen that poses a major challenge to clinical treatment. Bacteriophages encode diverse regulatory proteins that can alter host cellular processes during infection. Here we show that the ΦPA1019-encoded transcription factor Gp53 regulates bacterial physiology by targeting key host pathways. Gp53 expression reduces bacterial growth, motility, and biofilm formation, and causes filamentous cell morphology. Transcriptomic analysis reveals that Gp53 alters the expression of 3,342 genes, including genes involved in cell division and virulence-associated processes. DNA-binding assays demonstrate that Gp53 directly represses promoters of key host genes, including *ftsZ*, *fleQ*, *pslA*, and *amrZ*. Gp53-mediated repression of *ftsZ* disrupts Z-ring formation and inhibits cell division, while *ftsZ* expression restores these defects. Structural and mutational analyses identify residues important for Gp53-DNA interaction. Together, these findings reveal a phage-encoded mechanism that coordinately interferes with bacterial cell division and virulence-associated pathways, providing mechanistic insights into host transcriptional regulation during phage infection and expanding our understanding of phage-host interactions.

**Figure:**
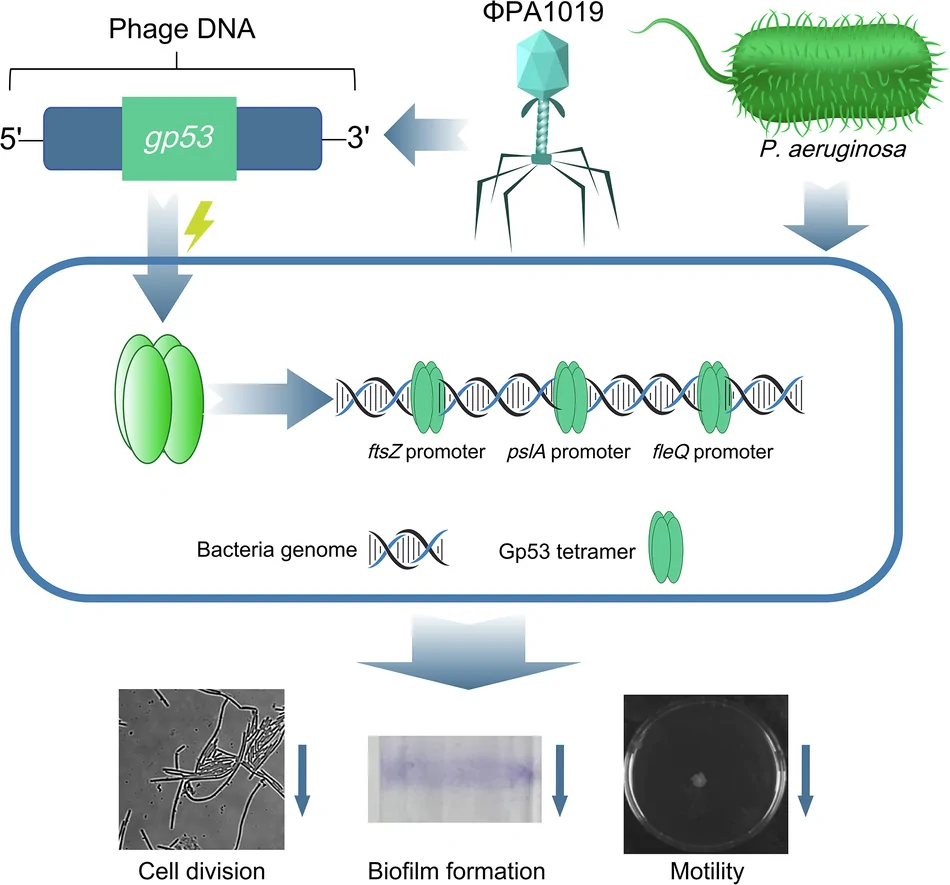

## Introduction

*Pseudomonas aeruginosa* is a Gram-negative opportunistic pathogen that is widely distributed in diverse natural environments. It exhibits remarkable environmental adaptability and multidrug resistance, enabling it to thrive under various stress conditions. As a major cause of hospital-acquired infections, *P. aeruginosa* is responsible for both acute and chronic infections that pose a serious threat to immunocompromised patients^1–3^. The increasing prevalence of antibiotic resistance in *P. aeruginosa* has rendered clinical treatment increasingly difficult, underscoring the urgent need for alternative antimicrobial strategies.

The extensive use of antibiotics have further accelerated the emergence of multidrug-resistant bacterial strains. Consequently, bacteriophage therapy has gained renewed attention as a complementary or alternative approach to antibiotic treatment^4,5^. Phage-based interventions have shown notable efficacy against bacterial infections, particularly in chronic lung, wound, and bloodstream infections^6,7^. In patients with cystic fibrosis, phages can penetrate bacterial biofilms, effectively reducing bacterial load and improving lung function^7^. Moreover, personalized phage therapy has demonstrated therapeutic potential for infections caused by drug-resistant strains. For instance, Ferry et al. reported the successful clearance of a spinal abscess using phage therapy in combination with surgical treatment^8^.

The mechanisms underlying phage-host interactions primarily depend on the specific recognition and regulation of host targets by phage-encoded proteins. These proteins modulate host physiological processes through diverse molecular strategies. For example, the phage proteins Aqs1, PIT2, and Qst target quorum-sensing (QS) regulators such as LasR or PqsD in *P. aeruginosa*, thereby altering host virulence and influencing phage infection efficiency^9–11^. Similarly, phage-encoded proteins YIPs and Dap1 interact with the host diguanylate cyclase YfiN and phosphodiesterase DipA, respectively, to enhance c-di-GMP synthesis and promote biofilm formation^12,13^. The phage protein PIT4 attenuates host virulence by modulating multiple two-component regulatory systems in *P. aeruginosa*^14^. Phage proteins can also directly interfere with host replication and transcription. For instance, the early-expressed protein Igy of phage LUZ24 mimics DNA structure to bind the host gyrase GyrB, thereby blocking bacterial replication and ultimately leading to cell death^15^. In addition, several phage proteins inhibit host growth by targeting the cell division machinery. Gp0.4 from phage T7 and Hdi from phage T5, for example, directly interact with FtsZ, disrupting Z-ring assembly and cell division in *Escherichia coli*^16,17^. Collectively, these findings illustrate that phage-encoded regulatory proteins can rapidly sense and manipulate host physiological states, presumably to facilitate phage propagation.

This study focuses on Gp53, a ribbon-helix-helix (RHH) DNA-binding protein encoded by *P. aeruginosa* phage ΦPA1019, whose homologs, such as Arc and AmrZ, play pivotal roles in the physiological regulation of phages and bacteria, respectively. The bacteriophage P22 Arc repressor controls the switch between lysogenic and lytic cycles by binding to phage operator DNA sequences^18,19^. In *P. aeruginosa*, AmrZ functions as a global transcription factor that regulates the expression of virulence-associated genes involved in flagellar biosynthesis, biofilm formation, and motility^20–22^. In this work, we elucidated the molecular mechanism by which Gp53 regulates cell division and pathogenicity in *P. aeruginosa* PAO1 through integrated multi-omics and molecular biology approaches. Gp53 directly binds to the promoters of key genes, including *ftsZ*, *fleQ*, *pslA*, and *amrZ*, thereby repressing their transcription. This repression leads to inhibition of cell division, motility, biofilm formation, and virulence. Our findings identify Gp53 as a previously uncharacterized phage-encoded regulator that directly targets key host pathways controlling cell division and pathogenicity, expanding the repertoire of mechanisms used by phages to manipulate bacterial physiology.

## Results

### RT-qPCR validation of early-expressed genes in bacteriophage ΦPA1019

Early-expressed bacteriophage genes are activated at the initial stage of infection and often encode regulatory proteins that modulate host gene expression and reshape bacterial regulatory networks to facilitate phage replication^23^. In this study, a *P. aeruginosa* phage designated ΦPA1019, was isolated from sewage samples. Whole-genome sequencing analysis revealed that the ΦPA1019 genome is 94,085 bp, which contains 132 open reading frames (ORFs). The phage exhibited efficient adsorption and a broad host lysis spectrum (Supplementary Table S3), suggesting potential for clinical and biocontrol applications against *P. aeruginosa* infections. To explore the roles of ΦPA1019 early-expressed genes in modulating host physiological processes, 12 candidate early genes were selected based on genome annotation (Supplementary Table S4). RT-qPCR analysis was performed using the late-stage capsid gene *gp5* as an internal reference to quantify transcriptional dynamics at 0, 5, 30, and 60 min post-infection (mpi) of *P. aeruginosa* PAO1. The results revealed significant upregulation of *gp46*, *gp47*, *gp53*, *gp60*, *gp63*, and *gp111* at 5 mpi, confirming their classification as early-expressed genes. Among them, *gp53* displayed the highest expression level, with a 4.4-fold increase compared with the control gene (Supplementary Fig. S1).

### Overexpression of *gp53* reduces biofilm formation and bacterial motility and inhibits cell division of *P. aeruginosa* PAO1

**Fig. 1:**
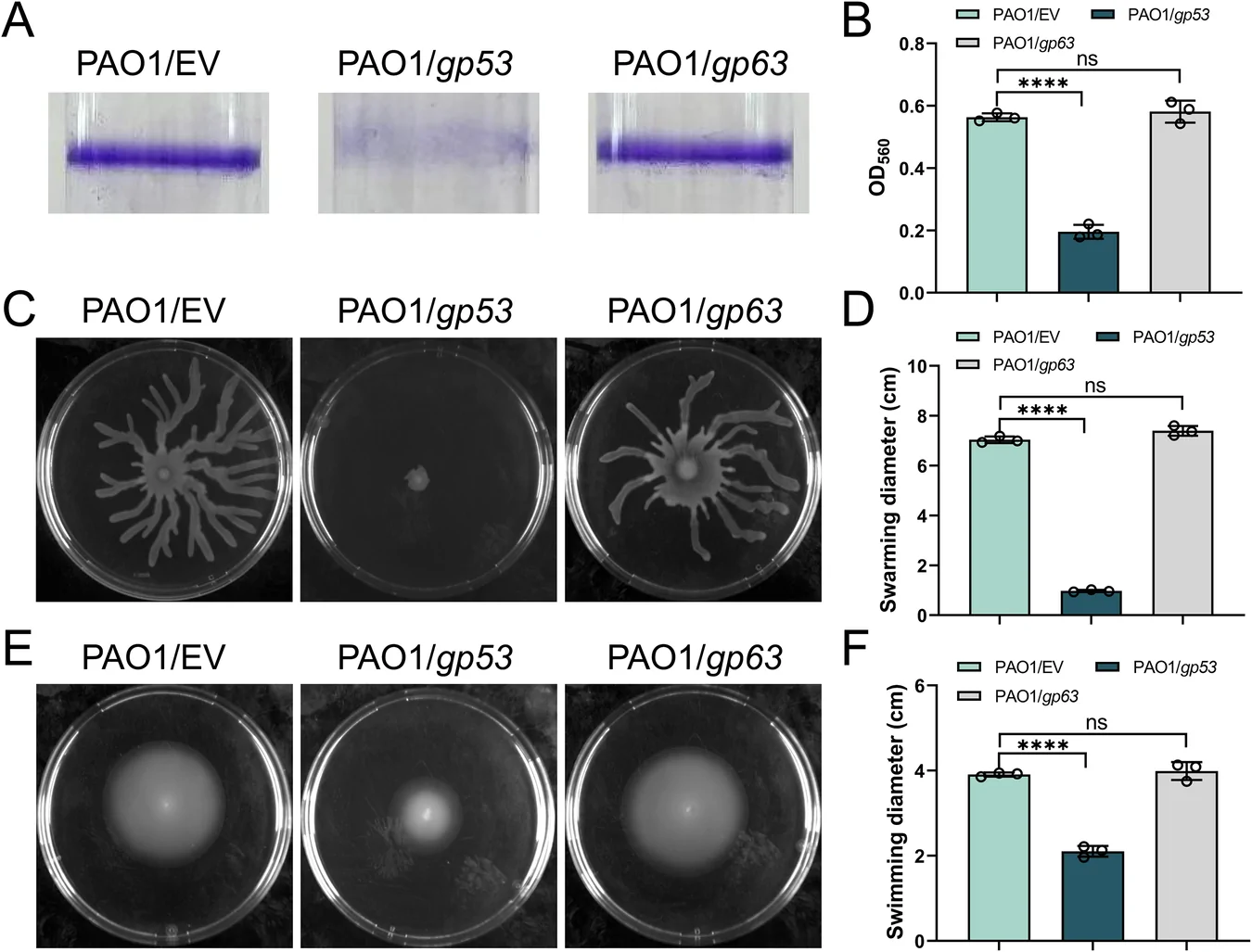
Overexpression of *gp53* reduces biofilm formation and motility in *P. aeruginosa* PAO1. **A** Visualization of biofilm formation. Strains were inoculated into LB medium containing 0.5 mM IPTG (1:100, v/v), and statically cultured at 25 °C for 12 h. Formed biofilms were stained with 0.1% crystal violet. **B** Quantification of biofilm formation. Stained biofilms were solubilized with 30% acetic acid, and absorbance was measured at 560 nm. **C** Swarming motility and **E** Swimming motility. Overnight cultures as 2 µL were inoculated onto the corresponding plates containing 0.5 mM IPTG and incubated at 37 °C or 30 °C for 12 h, respectively. Motility phenotypes were photographed using a Tanon 1600 imaging system. **D**, **F** Measurement of motility zone diameters. EV represents the empty vector pMMB67EH in this and subsequent experiments. Data represent mean ± standard deviation (SD) of three independent experiments (*n* = 3). ****P < 0.0001, ns, not significant, based on two-tailed Student′s *t*-test.

Motility and biofilm formation are key determinants of *P. aeruginosa*’s dispersal capacity and antibiotic resistance. To explore the function of early-expressed genes from phage ΦPA1019, each candidate gene was overexpressed in *P. aeruginosa* PAO1 using plasmid pMMB67EH under the control of the IPTG-inducible *tac* promoter. Among these genes, *gp53* overexpression markedly suppressed biofilm formation and bacterial motility. As shown in Fig. 1, biofilm biomass in the *gp53*-overexpressing strain was approximately 3-fold lower than that of wild-type PAO1, and both swarming and swimming motilities were substantially reduced. In contrast, overexpression of *gp63* showed no significant effect on these phenotypes. These findings indicate that *gp53* expression is associated with reduced biofilm formation and motility, suggesting a broader impact on bacterial physiology and adaptability.

**Fig. 2:**
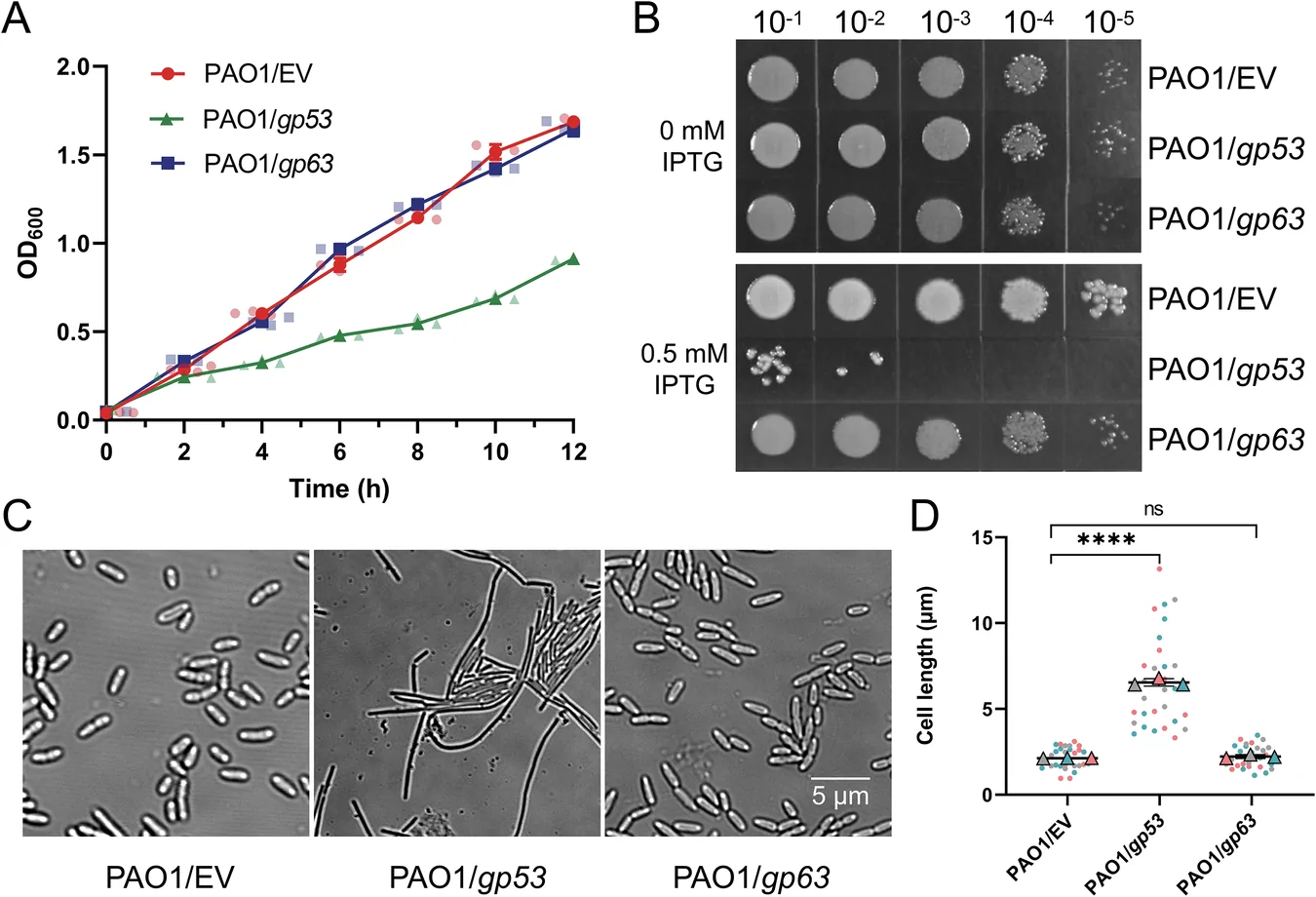
Overexpression of *gp53* inhibits bacterial growth and cell division in *P. aeruginosa* PAO1. **A** Bacterial growth curves. Strains were inoculated into LB medium containing 0.5 mM IPTG and cultured with shaking at 37 °C. Optical density at 600 nm was measured every 2 h. Data represent mean ± SD from three biological replicates (*n* = 3). **B** Agar spot assay for growth assessment. Cultures grown for 6 h were serially diluted, and 2.5 μL of each dilution was spotted onto LB agar plates, followed by incubation at 37 °C for 24 h. **C** Cell morphology observation by laser confocal microscopy after 6 h of induction. All images were captured under identical magnification and imaging conditions. Scale bar, 5 μm. **D** Cell length quantification of *P. aeruginosa* strains shown in (**C**). Each dot represents an individual cell, with colors indicating three independent biological replicates (10 cells measured per replicate). Large triangles indicate the mean cell length of each replicate, while horizontal lines and error bars represent the mean ± SD of three biological replicates. Statistical analyses were performed using biological replicates as the experimental unit (*n* = 3) by one-way ANOVA followed by Dunnett’s multiple-comparisons test. ****P < 0.0001, ns, not significant.

To further assess the effects of *gp53* overexpression, bacterial growth dynamics and cell morphology were examined. Growth curve analysis revealed that the proliferation rate of PAO1 pMM-*gp53* was markedly lower than that of the wild-type strain (Fig. 2A). Consistently, agar spot assays confirmed a significant growth inhibition in the *gp53*-overexpressing strain (Fig. 2B). Besides, Laser confocal microscopy showed that *gp53* overexpression induced a striking alteration in cell morphology, with cells exhibiting pronounced elongation and filamentous structures after 6 h of induction (Fig. 2C, D).

**Fig. 3:**
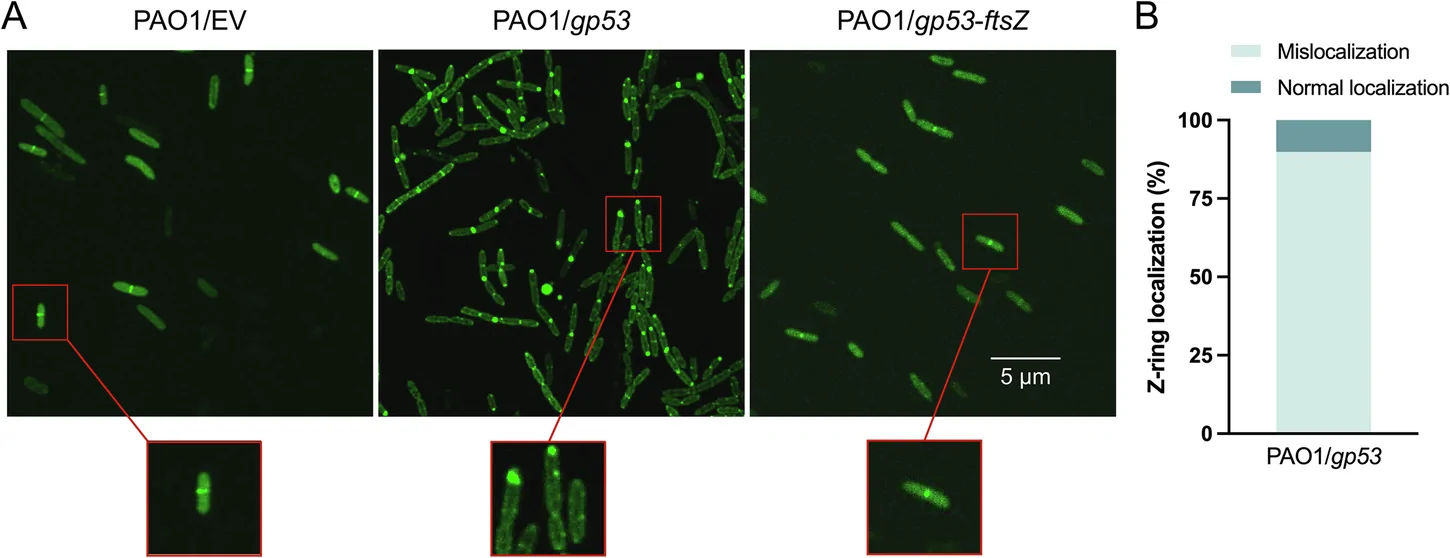
Effect of *gp53* overexpression on Z-ring localization in *P. aeruginosa* PAO1. **A** Representative fluorescence microscopy images showing ZapA-GFP localization in different *P. aeruginosa* strains. Cells were inoculated into LB medium containing 0.5 mM IPTG at a 1:100 dilution and induced for 2 h before imaging. The localization of ZapA-GFP was visualized using a laser confocal microscope. Scale bar, 5 μm. **B** Quantification of Z-ring localization patterns. Cells exhibiting non-central or aberrantly positioned Z-rings were classified as having mislocalized Z-rings. The y-axis represents the percentage of cells displaying normal or mislocalized Z-ring positioning, *N* = 80. PAO1/EV harboring pMMB67EH and pME-*zapA*-GFP, PAO1/*gp53* harboring pMM-*gp53* and pME-*zapA*-GFP, PAO1/*gp53*-*ftsZ* harboring pMM-*gp53*-*ftsZ* and pME-*zapA*-GFP.

In *P. aeruginosa*, cell division depends on the formation of the Z-ring, primarily composed of FtsZ and its associated proteins such as ZapA^24^. Because direct fluorescent labeling of FtsZ can interfere with its native function, the Z-ring localization was instead visualized using ZapA fused with GFP, which is recruited by FtsZ during cytokinesis. In wild-type PAO1, ZapA-GFP fluorescence was predominantly localized at the midcell, corresponding to normal Z-ring assembly. In contrast, in the *gp53* overexpression strain, ZapA-GFP signals were dispersed or mislocalized, indicating disruption of proper Z-ring positioning (Fig. 3). Collectively, these results demonstrate that Gp53 interferes with the physiological homeostasis of *P. aeruginosa* by impairing cell growth and division through disturbance of Z-ring assembly and spatial organization.

### RNA-seq and RT-qPCR analysis of *gp53* overexpressing strain

**Fig. 4:**
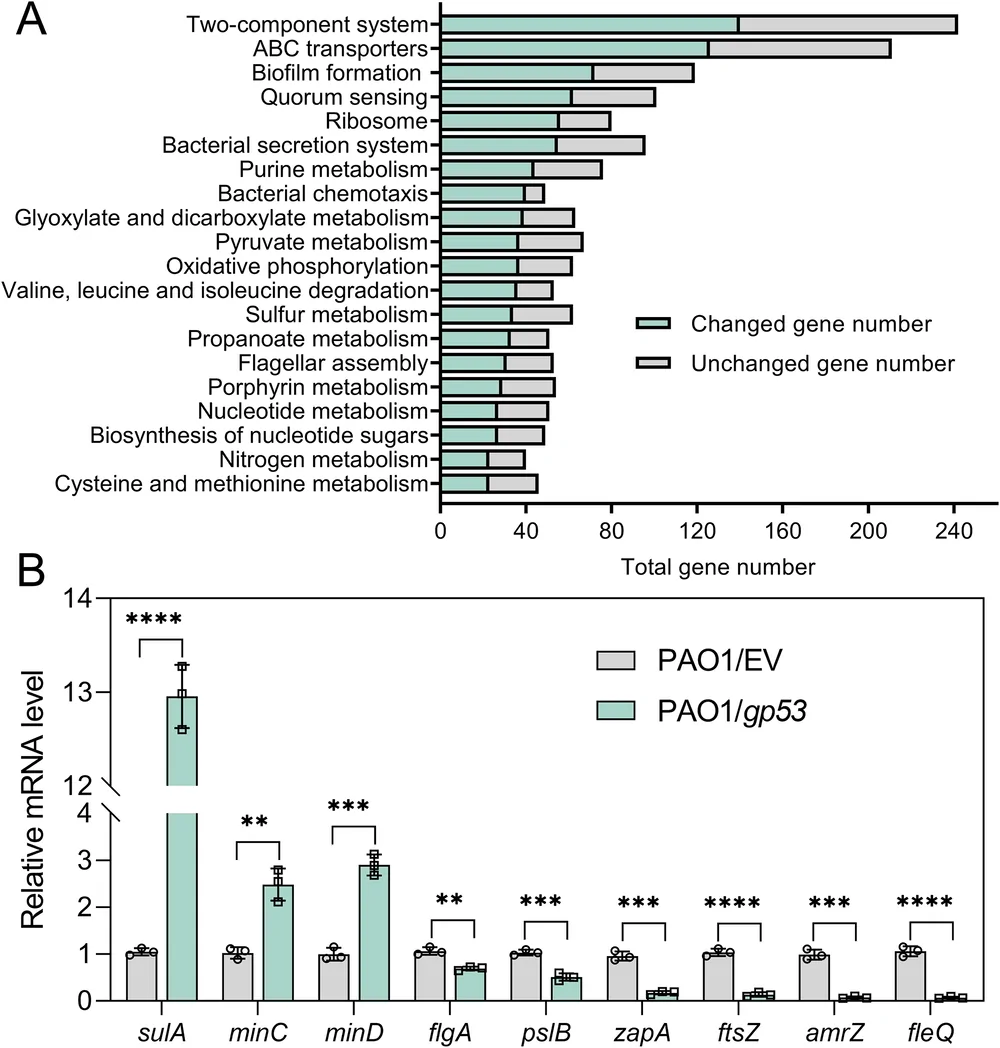
RNA-seq and RT-qPCR analysis of *gp53* overexpressing strains. **A** KEGG enrichment of DEGs identified in the RNA-seq analysis. The y-axis represents the top 20 enriched pathways. The x-axis indicates the total number of genes within each pathway. **B** RT-qPCR validation of gene expression. Strains were cultured to OD_600_ = 0.6, and total RNA was extracted. Gene expression levels were normalized to *rpsL* expression. Data represent mean ± SD from three biological replicates (*n* = 3). **P < 0.01, ***P < 0.001, and ****P < 0.0001, determined by two-tailed Student′s *t*-test. Exact *P* values are shown as follows: *sulA*, *P* = 0.00000047; *minC*, *P* = 0.002265; *minD*, *P* = 0.00023; *flgA*, *P* = 0.002033; *pslB*, *P* = 0.000814; *zapA*, *P* = 0.000196; *ftsZ*, *P* = 0.000074; *amrZ*, *P* = 0.000132; and *fleQ*, *P* = 0.000092.

Based on the above findings, *gp53* overexpression significantly affected multiple phenotypic traits of *P. aeruginosa*. Sequence analysis revealed that *gp53* encodes a 126-amino-acid RHH DNA-binding protein (Supplementary Fig. S2). Other members of this family include the Arc repressor of bacteriophage P22^18,19^ and the transcriptional regulator AmrZ in *P. aeruginosa*^20–22^. To elucidate the regulatory network governed by Gp53, transcriptome sequencing analysis was performed on *P. aeruginosa* PAO1 strains harboring either the control plasmid pMMB67EH or pMM-*gp53*. A total of 3342 differentially expressed genes (DEGs) were identified using the criteria of |log2 fold change | ≥1 and adjusted *P*-value < 0.05, including 1642 upregulated and 1700 downregulated genes in the *gp53* overexpression strain (Supplementary Fig. S3). KEGG pathway enrichment analysis revealed significant enrichment of DEGs in 20 biological pathways, notably the two-component system (140 genes), ABC transport (126 genes), biofilm formation (72 genes), quorum sensing (62 genes), and flagellar assembly (31 genes) (Fig. 4A).

Among these DEGs, expression of core cell division genes associated with Z-ring formation, such as *ftsZ*, *ftsA*, and *zapA* was markedly suppressed, whereas the expression of division inhibitors *sulA* and *minC/D/E* was upregulated (Supplementary Table S5). Similarly, genes involved in biofilm synthesis (*pslB/C*, *amrZ*, *algC*, *cdrB*) and motility (*flgA/B/C*, *fleQ*, *motC/D*, and *pilA/U*) were significantly downregulated. Moreover, virulence-associated genes including *lasR/rhlR*, *exsC*, *phzA1/A2*, and *rhlA/B* exhibited reduced transcriptional levels. Collectively, these results indicate that Gp53 functions as a global transcriptional regulator that modulates diverse cellular processes in *P. aeruginosa*, including cell division, biofilm formation, motility, and virulence.

To validate the RNA-seq results, nine representative genes involved in key physiological processes were selected for RT-qPCR analysis in PAO1 pMMB67EH and PAO1 pMM-*gp53*. The housekeeping gene *rpsL* gene was used as an internal control. As shown in Fig. 4B, the RT-qPCR results were consistent with the RNA-seq data. Overexpression of *gp53* in PAO1 significantly reduced the expression of genes involved in cell division Z-ring, biofilm synthesis, and motility, such as *ftsZ*, *pslB*, and *fleQ*, while upregulating genes related to division inhibition, including *sulA* and *minC/D* (Fig. 4B).

To assess whether the IPTG-induced expression system produced physiologically relevant levels of *gp53*, we further compared *gp53* transcript abundance under IPTG induction and during ΦPA1019 infection by RT-qPCR. Expression levels were normalized to the housekeeping gene *rpsL*. The results showed that *gp53* expression induced by 0.5 mM IPTG was approximately 2-fold higher than that detected at 5 min post-infection during ΦPA1019 infection (Supplementary Fig. S4). Although IPTG induction resulted in moderately elevated transcript levels, the expression levels observed under the two conditions were of the same order of magnitude, supporting the suitability of the IPTG-inducible system for functional characterization of Gp53.

### DAP-seq analysis identifies the DNA-binding sites of Gp53

**Fig. 5:**
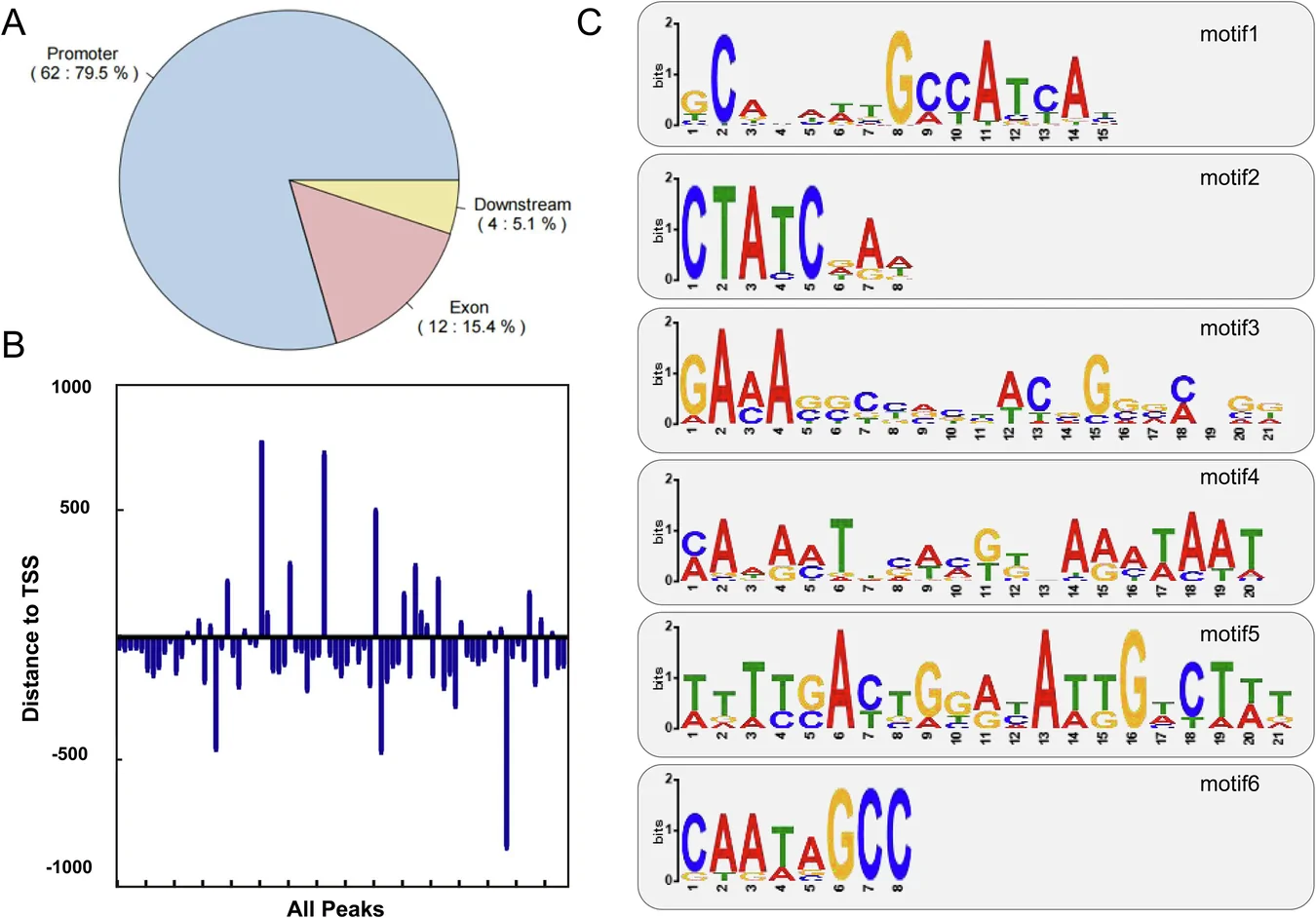
DAP-seq analysis identifies the DNA-binding sites of Gp53. **A** Distribution of Gp53 binding peaks across the *P. aeruginosa* PAO1 genome. **B** Binding peak distribution, showing 76.92% enrichment within the core promoter regions, specifically between −200 to +200 bp relative to the transcription start sites (TSSs). **C** De novo motif prediction identified six potential Gp53-binding motifs, including a high-confidence motif (motif1, *E*-value = 1.8 × 10^−113^).

To identify the DNA-binding sites of Gp53 in the *P. aeruginosa* PAO1 genome, a genome-wide DAP-seq analysis was performed. The result revealed that Gp53 specifically bound to 78 DNA regions within the PAO1 genome, with 62 of these sites (79.5%) located in core regulatory regions of gene promoters (Fig. 5A). Target genes included biofilm formation genes *amrZ*, *pslA* and *pelA*, motility related genes *flgA* and *fleQ*, virulence genes *phzA2* and *wzz2*; c-di-GMP metabolic enzyme genes *mucR* and *roeA*, and bacterial secretion system genes *tssA1* and *hsiA2* (Supplementary Table S6), confirming that Gp53 directly regulates key pathogenicity-related genes in *P. aeruginosa* at the transcriptional level. Binding site distribution analysis showed that 76.92% of the binding peaks were concentrated in the −200 to +200 bp region upstream of the transcription start site (Fig. 5B). Motif analysis using the MEME Suite and HOMER identified six potential binding motifs (Fig. 5C), with motif1 displaying a very high confidence level (*E*-value of 1.8 × 10^−113^).

**Fig. 6:**
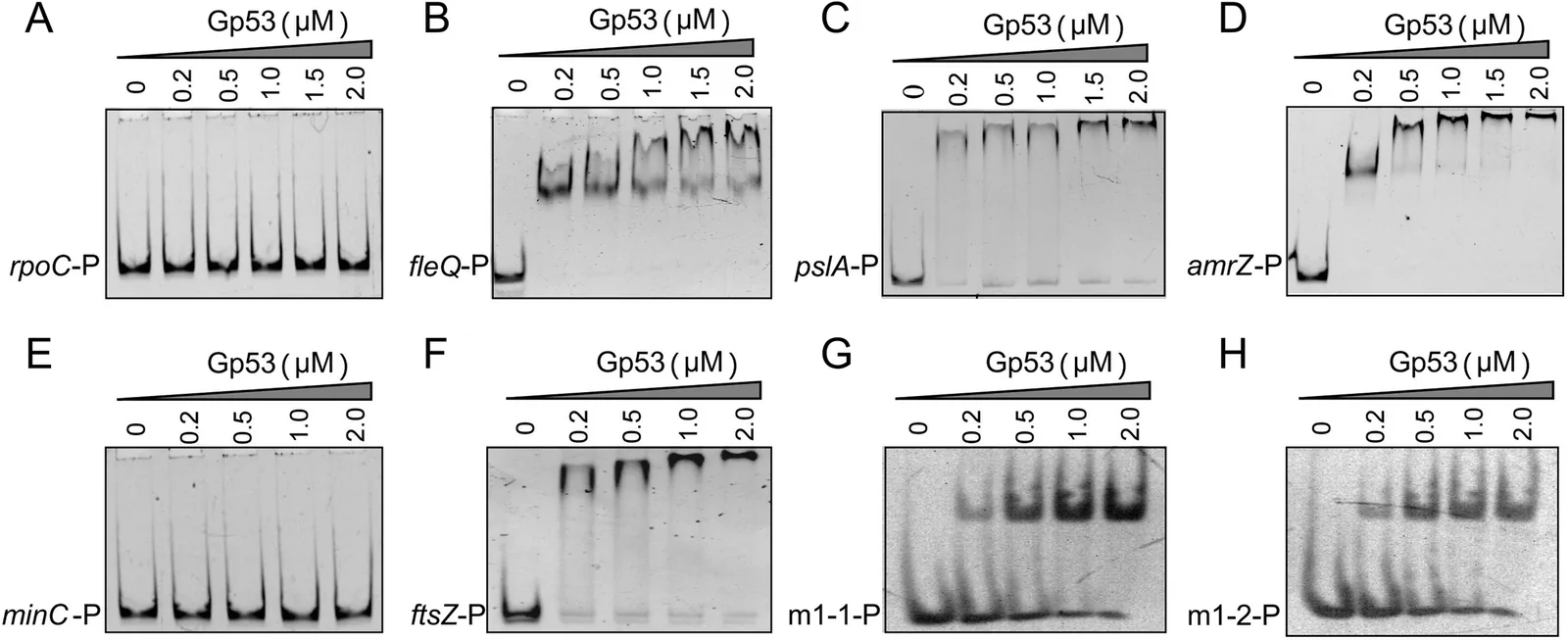
Gp53 binds to the selected promoter regions in vitro. **A**–**D** EMSA assays confirmed dose-dependent binding of Gp53 to *fleQ*, *pslA*, and *amrZ* promoters, with *rpoC* as a non-target control. **E**–**H** Specific binding of Gp53 to the full-length *ftsZ* promoter and two putative binding sites was confirmed, while no binding was observed with the *minC* promoter. Purified Gp53 protein was incubated with 50 ng promoter probes in 20 μL binding buffer. Gp53 protein was added in lanes 2–5/6. Lane 1 served as the negative control with no protein added.

To validate the DAP-seq findings, we performed EMSA assay to further confirm the DNA-binding ability of Gp53. Three promoter regions of Gp53 targeted genes including *fleQ*, *pslA*, and *amrZ* were selected for in vitro testing, with the *rpoC* promoter serving as a negative control. As shown in Fig. 6A–D, Gp53 bound to the promoters of *fleQ*, *pslA*, and *amrZ* in a dose-dependent manner. In contrast, the *rpoC* promoter showed no binding even at the highest protein concentration. These results confirm that Gp53 directly regulates the expression of biofilm and motility-related genes in *P. aeruginosa*, thereby inhibiting both biofilm formation and motility.

RNA-seq analysis revealed a significant upregulation of *minC*, a negative regulator of cell division, while the expression of *ftsZ*, a key gene in Z-ring assembly, was downregulated. Although DAP-seq did not detect binding of Gp53 to the *ftsZ* or *minC* promoters, EMSA assays demonstrated that Gp53 could specifically bind to the *ftsZ* promoter, but not the *minC* promoter (Fig. 6E, F). Sequence alignment of the 298 bp *ftsZ* promoter region with the forward and reverse sequences of the high-confidence motif1 identified two potential Gp53 binding sites, designated m1-1 and m1-2 (Supplementary Fig. S5). Notably, m1-1 was located immediately adjacent to the −10 box within the *ftsZ* promoter region. EMSA analysis confirmed that Gp53 specifically binds to both regions (Fig. 6G, H). Taken together, these findings provide compelling evidence that Gp53 directly binds to the *ftsZ* promoter and inhibits *ftsZ* transcription, thereby disrupting the host cell division process.

### Co-expression of *ftsZ* and *gp53* restores cell division in *P. aeruginosa* PAO1

**Fig. 7:**
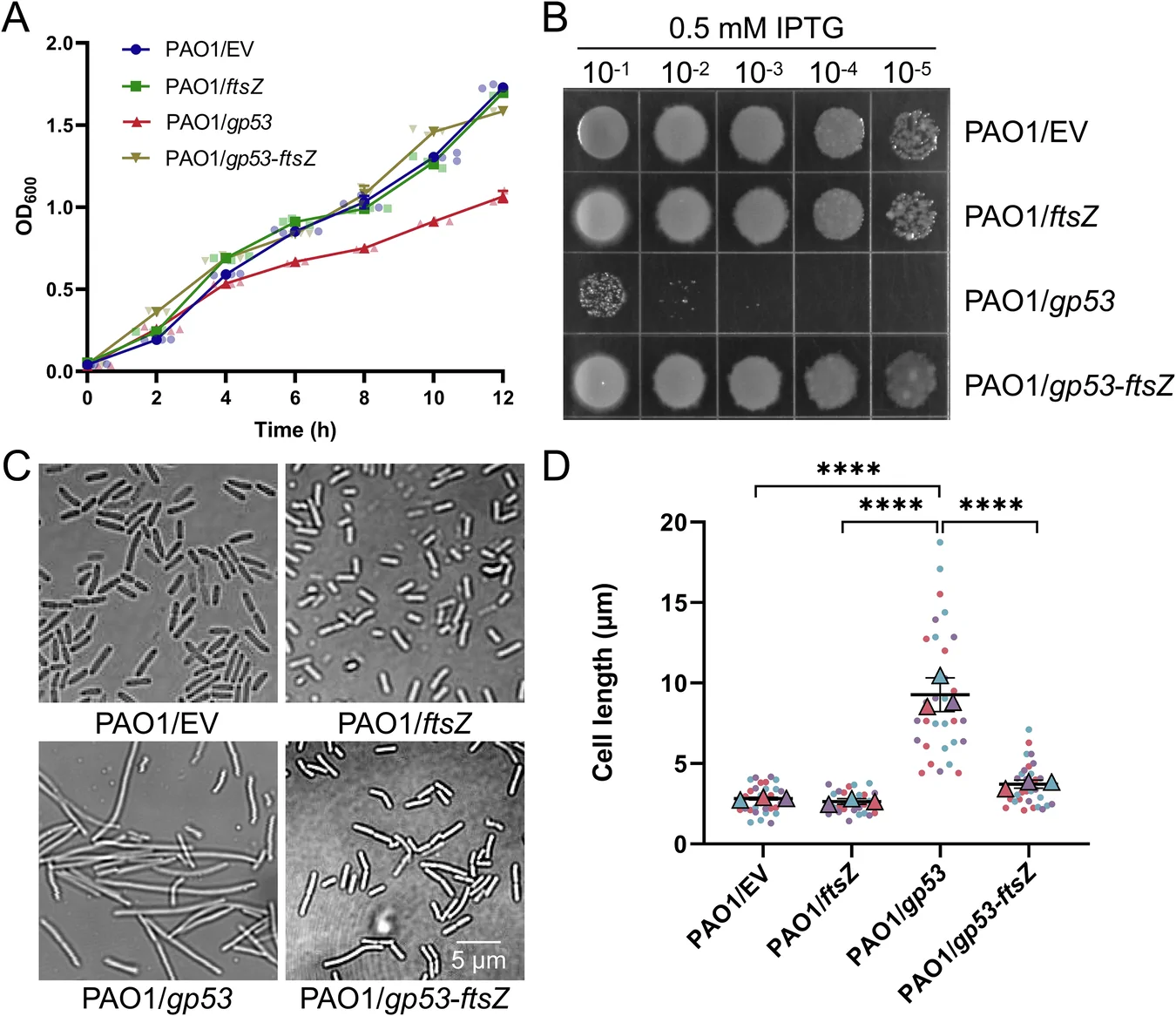
Co-expression of *ftsZ* and *gp53* restores cell division in *P. aeruginosa* PAO1. **A** Bacterial growth was determined by measuring OD_600_ using a spectrophotometer. Strains were inoculated into LB medium containing 0.5 mM IPTG, and samples were taken every 2 h to measure OD_600_. Data represent mean ± SD from three biological replicates (*n* = 3). **B** Agar spot assays to assess bacterial growth. Strain cultures after 6 h of growth were serially diluted, spotted on LB plates, and cultured at 37 °C for 24 h. **C** Cell morphology was observed and photographed using a laser confocal microscope after 6 h of growth, and all images were taken at the same magnification. Scale bar, 5 μm. **D** Cell length quantification of *P. aeruginosa* strains shown in (**C**). Each dot represents an individual cell, with colors indicating three independent biological replicates (10 cells measured per replicate). Large triangles indicate the mean cell length of each replicate, while horizontal lines and error bars represent the mean ± SD of three biological replicates. Statistical analyses were performed using biological replicates as the experimental unit (*n* = 3) by one-way ANOVA followed by Dunnett’s multiple-comparisons test. ****P < 0.0001. PAO1/EV harboring pMMB67EH and pME6032, PAO1/*ftsZ* harboring pMMB67EH and pME-*ftsZ*, PAO1/*gp53* harboring pMM-*gp53* and pME6032, PAO1/*gp53*-*ftsZ* harboring pMM-*gp53* and pME-*ftsZ*.

The previous results confirmed the interaction between Gp53 and *ftsZ* promoter. To further investigate whether Gp53 regulates cell division through *ftsZ*, we co-overexpressed *ftsZ* and *gp53* in PAO1 and evaluated bacterial growth and cell morphology. The results showed that co-expression of *gp53* and *ftsZ* in PAO1 restored the growth inhibition (Fig. 7A, B), filamentous phenotype (Fig. 7C, D), and Z-ring mislocalization (Fig. 3A) induced by Gp53 overexpression, indicating that Gp53 influences cell division and bacterial growth by inhibiting *ftsZ* transcription. However, co-expression of *ftsZ* did not reverse the decrease in biofilm production or motility defects associated with Gp53 overexpression (Supplementary Fig. S6), suggesting that the regulation of biofilm and motility by Gp53 occurs through a mechanism independent of cell division. These findings, in conjunction with RNA-seq and DAP-seq data, demonstrate that Gp53 directly inhibits the transcription of key pathogenicity-related genes such as *ftsZ*, *fleQ*, *pslA*, and *amrZ*, confirming its role as a multi-target global transcriptional regulator.

### Structural modeling and mutational analyses identify key amino acid residues essential for the Gp53-DNA interaction

Protein sequence alignment showed that Gp53 contains a conserved RHH DNA-binding domain shared with Arc and AmrZ, with higher similarity observed between Gp53 and AmrZ protein (Supplementary Fig. S2). Previous studies have shown that both Arc and AmrZ bind DNA as tetramers^25–28^, suggesting that Gp53 may adopt a similar oligomeric state. Structural modeling and docking analysis further indicated that key N-terminal residues, including Arg15 and Asn36, may participate in DNA binding through hydrogen bond formation with the *ftsZ* promoter region (Supplementary Fig. S7, Supplementary Table S7).

Based on the above results, four amino acid residues, Arg15, Arg21, Met35, and Asn36 in β-sheet, α-helix A, or α-helix B of Gp53 protein were selected for site-directed mutagenesis to assess their role in DNA binding by the Gp53 (Supplementary Fig. S2). Protein expression analysis showed that the four mutant proteins, Gp53^R15A^, Gp53^R21A^, Gp53^M35A^, and Gp53^N36A^, exhibited expression levels and stability comparable to the wild-type Gp53 (Supplementary Fig. S8). EMSA assays were then performed to evaluate their DNA-binding activity. As shown in Supplementary Fig. S9, wild-type Gp53 induced a clear shift of the *ftsZ* promoter DNA, whereas the four mutants largely lost DNA-binding ability. Notably, Gp53^R21A^ retained weak binding to the *ftsZ* promoter only at a high concentration of 1.5 μM.

**Fig. 8:**
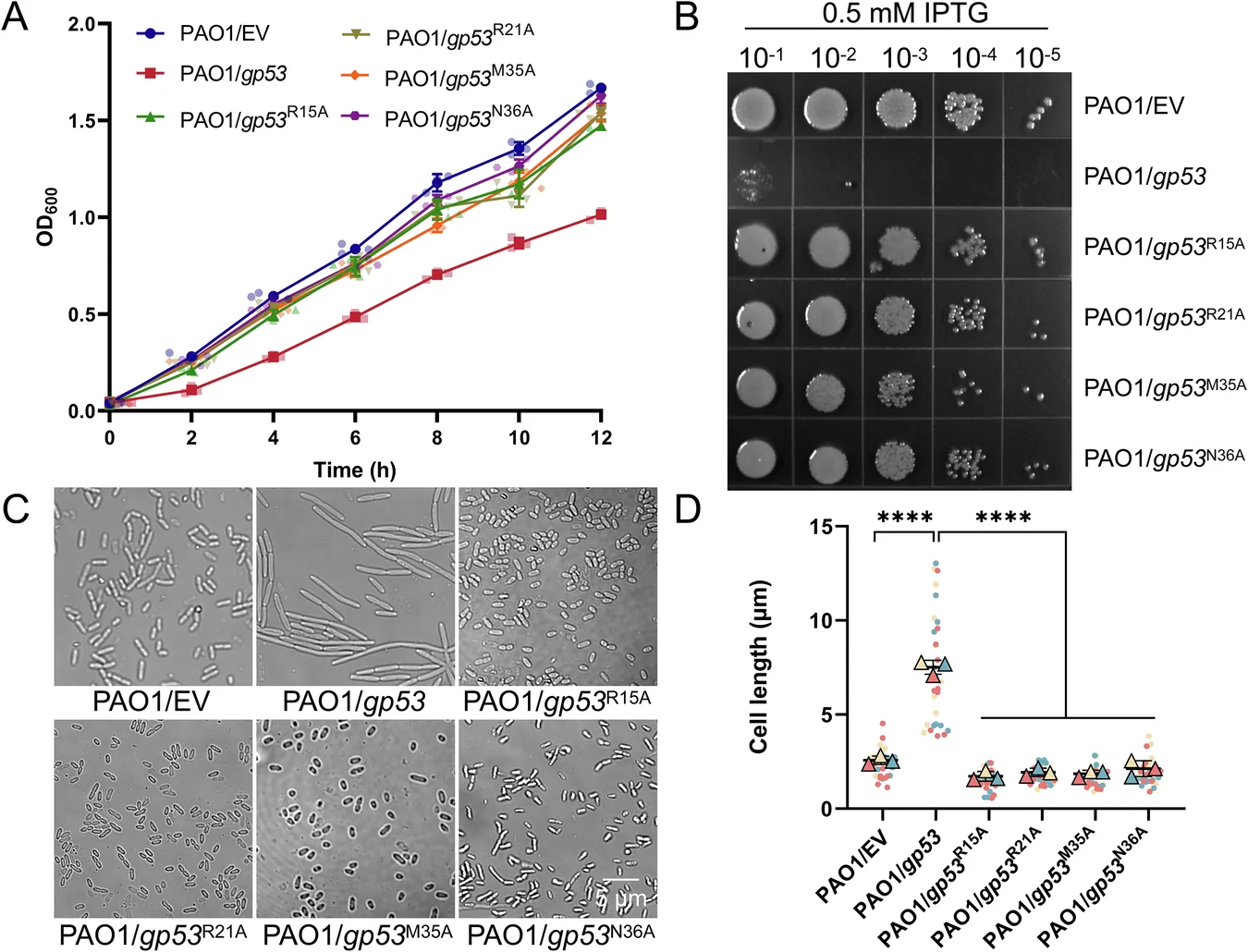
Effects of Gp53 mutations on bacterial growth and cell division in *P. aeruginosa* PAO1. **A** Bacterial growth was measured by OD_600_ using a spectrophotometer. Strains were inoculated into LB medium containing 0.5 mM IPTG, and samples were taken every 2 h to monitor OD_600_. Data represent mean ± SD from three biological replicates (*n* = 3). **B** Agar spot assays to assess bacterial growth. After 6 h of culture, strains were serially diluted, spotted on LB plates, and incubated at 37 °C for 24 h. **C** Cell morphology was observed and photographed by a laser confocal microscope after 6 h of growth, and all images were taken at the same magnification. Scale bar, 5 μm. **D** Cell length quantification of *P. aeruginosa* strains shown in (**C**). Each dot represents an individual cell, with colors indicating three independent biological replicates (10 cells measured per replicate). Large triangles indicate the mean cell length of each replicate, while horizontal lines and error bars represent the mean ± SD of three biological replicates. Statistical analyses were performed using biological replicates as the experimental unit (*n* = 3) by one-way ANOVA followed by Dunnett’s multiple-comparisons test. ****P < 0.0001.

Furthermore, the four site-directed Gp53 mutants were overexpressed in *P. aeruginosa* PAO1 and the resulting phenotypes were assessed. The result showed that mutations at Arg15, Arg21, Met35, and Asn36 largely restored bacterial growth and cell morphology to wild-type levels (Fig. 8). These findings are consistent with the EMSA results, confirming that these residues are critical for Gp53 binding to the *ftsZ* promoter DNA. Mutation of these amino acids not only abolished the DNA-binding ability of Gp53 but also relieved its inhibitory effects on bacterial growth and cell division. In addition, the defects in biofilm formation and motility caused by wild-type Gp53 were largely abolished in all four mutant strains (Supplementary Fig. S10). These results further demonstrate that Arg15, Arg21, Met35, and Asn36 are essential for the regulatory activity of Gp53 and support the conclusion that the inhibitory effects of Gp53 on host physiology depend on its DNA-binding ability.

## Discussion

*P. aeruginosa*, a major opportunistic pathogen responsible for nosocomial infections, poses a serious threat to individuals with weakened immune systems^29^. With the global emergence and dissemination of carbapenem-resistant *P. aeruginosa* strains, conventional antibiotic therapies are reaching a critical bottleneck, emphasizing the necessity of developing innovative antibacterial approaches^30,31^. Bacteriophage therapy has attracted increasing attention as a promising approach for targeted pathogen eradication due to its high specificity and efficiency^5,8^.

Phage early-expressed genes play pivotal roles during host infection, participating in processes such as suppression of host defenses, hijacking of cellular resources, and regulation of phage gene expression^32^. For instance, the early gene *gp0.7* of T7 phage targets the host RNA polymerase to inhibit bacterial transcription and thereby facilitate phage gene expression^33^. Similarly, the N protein of λ phage promotes transcriptional read-through via an anti-termination mechanism, ensuring efficient progression of the infection cycle^34^. In this study, fifteen potential early-expressed genes of ΦPA1019 were screened. Among them, overexpression of *gp53*, which encodes a RHH DNA-binding transcription factor, in *P. aeruginosa* PAO1 markedly altered host phenotypes, including reduced biofilm formation and motility (Fig. 1), as well as the induction of filamentous cell morphology (Fig. 2). These findings indicate that Gp53 interferes with host cell division and virulence factor expression, thereby exerting multifaceted regulatory effects on *P. aeruginosa* physiology.

To elucidate the global regulatory mechanism of Gp53, RNA-seq analysis was performed, revealing that its overexpression markedly influenced genes associated with cell division, biofilm formation, motility, and virulence (Fig. 4). Among the differentially expressed genes, those involved in Z-ring assembly, including *ftsZ*, *ftsA*, and *zapA*, were significantly downregulated, whereas cell division inhibitors such as *minC/D/E* and *sulA* were upregulated. Additionally, genes related to biofilm, motility, and the QS system were broadly repressed (Supplementary Table S5). DAP-seq analysis further identified 78 Gp53-binding sites across the genome, with 79.5% located in core promoter regions (Fig. 5A). These targets included genes associated with biofilm formation, motility, virulence, c-di-GMP metabolism, and bacterial secretion systems (Supplementary Table S6). Motif prediction identified six distinct Gp53-binding motifs (Fig. 5C). Together, the RNA-seq and DAP-seq data demonstrate that Gp53 acts as a global transcriptional regulator that orchestrates a complex regulatory network through multiple pathways.

Unlike the phage proteins Gp0.4 and Hdi, which inhibit cell division in *E. coli* by directly interacting with the FtsZ protein^16,17^, Gp53 suppresses cell division in *P. aeruginosa* PAO1 by downregulating *ftsZ* expression. EMSA analysis confirmed that Gp53 directly binds to the *ftsZ* promoter (Fig. 6F). Sequence alignment with the high-confidence DAP-seq motif1 revealed two potential Gp53-binding regions within the *ftsZ* promoter, and EMSA further verified that Gp53 specifically binds to both sites, thereby directly repressing *ftsZ* transcription (Fig. 6G, H). Moreover, fluorescence imaging of the cell division Z-ring showed that in the *gp53*-overexpressing strain, the Z-ring was markedly displaced from its normal mid-cell position, indicating that Gp53 interferes with proper Z-ring assembly by repressing *ftsZ* expression (Fig. 3). Co-expression of *gp53* and *ftsZ* restored normal cell division (Fig. 7), but did not rescue the biofilm formation or motility defects (Supplementary Fig. S6). Given that Gp53 can also directly bind to the promoters of biofilm and motility-related genes such as *fleQ*, *pslA*, and *amrZ* (Fig. 6B–D), these phenotypes are likely independent of the division inhibition pathway. Collectively, these findings suggest that Gp53 functions as a global transcriptional repressor that directly downregulates multiple gene networks governing cell division, biofilm formation, motility, and virulence in *P. aeruginosa* PAO1.

We attempted to resolve the three-dimensional structure of Gp53 by X-ray crystallography; however, its strong tendency to aggregate made purification and crystallization difficult. Therefore, we employed the AlphaFold Server to predict the complex structure between Gp53 and the *ftsZ* promoter. Given that its homologous proteins, Arc and AmrZ, bind DNA as tetramers through their RHH domains^27,28^, Gp53 was hypothesized to interact with target promoters in a similar tetrameric configuration. Structural modeling supported this hypothesis and revealed several key amino acid residues involved in DNA recognition (Supplementary Fig. S7). Consistent with these predictions, site-directed mutagenesis confirmed four residues, Arg15, Arg21, Met35, and Asn36, within the N-terminal DNA-binding motif as critical for Gp53-DNA interaction. Mutation of these residues abolished DNA binding and alleviated the inhibitory effects of Gp53 on bacterial growth, cell division, biofilm formation, and motility (Supplementary Fig. S9, Fig. 8 and Supplementary Fig. S10). Interestingly, these corresponding amino acid residues have also been reported to participate in the promoter-binding process of the AmrZ protein^26^. Moreover, several target promoters of Gp53, including *fleQ*, *pslA*, *amrZ*, and *algD*, are also recognized by AmrZ^20,22,35,36^. However, our additional experiments demonstrated that the functions of Gp53 and AmrZ are not completely overlapping. Unlike Gp53, overexpression of *amrZ* did not result in comparable defects in bacterial growth, biofilm formation, or motility, nor did it rescue the phenotypes caused by *gp53* overexpression (Supplementary Fig. S11). These findings suggest that, despite their structural similarities and partially shared regulatory targets, Gp53 and AmrZ are functionally distinct regulators. Whether Gp53 directly competes with, cooperates with, or otherwise influences AmrZ-mediated regulation at shared promoters remains an intriguing question for future investigation.

In this study, we systematically elucidated the molecular mechanism by which the early-expressed RHH-type DNA-binding protein Gp53 from phage ΦPA1019 modulates cell division and pathogenicity in *P. aeruginosa*. Gp53 directly binds to the promoter regions of *ftsZ*, *fleQ*, *pslA*, and *amrZ*, thereby repressing the transcription of key genes involved in bacterial growth and division, biofilm formation, motility, and virulence. Collectively, these findings identify Gp53 as a phage-encoded transcriptional regulator that reprograms host gene expression at a global level. Such transcriptional and physiological reprogramming may facilitate efficient phage propagation by redirecting host metabolic and regulatory resources toward viral replication while simultaneously suppressing energy-intensive cellular processes. These results provide mechanistic insights into phage-mediated host regulation and expand our understanding of how phages manipulate bacterial physiology during infection.

## Methods

### Bacterial strains, media and plasmids

The strains, plasmids and primers used in this study were shown in Supplementary Tables S1 and S2. Strains were cultured in Luria-Bertani (LB) medium at 37 °C. For solid media, agar was added to a final concentration of 1.2%. Appropriate concentrations of antibiotics were added when necessary. *E. coli* strains were grown in LB medium supplemented with 100 µg/mL carbenicillin (Cb), 50 µg/mL kanamycin (Kn), and 10 µg/mL tetracycline (Tc), whereas *P. aeruginosa* strains were cultured in LB medium containing 300 µg/mL Cb and 70 µg/mL Tc.

### Isolation and purification of bacteriophage ΦPA1019

Bacteriophage ΦPA1019 was isolated from sewage samples using enrichment culture followed by plaque purification. Hospital wastewater samples were clarified by sedimentation, and the supernatants were incubated with exponentially growing *P. aeruginosa* PAO1 to enrich phages. After incubation, cultures were centrifuged and the supernatants were filtered through a 0.22 μm membrane. Phage presence was verified by spot assays on PAO1 lawns. Individual plaques were then picked and subjected to multiple rounds of dilution and double-layer agar plaque purification until uniform, well-defined plaques were obtained, yielding a purified phage ΦPA1019.

### Overexpressing genes in *P. aeruginosa*

The *gp53* gene from phage ΦPA1019 was overexpressed in *P. aeruginosa* PAO1 from plasmid pMMB67EH under the control of the IPTG-inducible *tac* promoter. The *gp53* gene was amplified by PCR using phage ΦPA1019 as a template. The PCR product was double digested and ligated into pMMB67EH to construct pMM-*gp53*. The resulting plasmid was introduced into PAO1 by electroporation. A similar approach was employed for overexpression of other genes. For co-expression experiments, the plasmid pME6032 was used to express *ftsZ* or *zapA*-*gfp* in the *gp53* overexpressing strains.

### Site-directed mutagenesis

Site-directed mutations in *gp53* were generated by overlap extension PCR as described previously^37^. Briefly, mutagenic primers carrying the desired nucleotide substitutions were used to amplify overlapping DNA fragments with PrimeStar^TM^ DNA polymerase (Takara, Japan). The resulting PCR fragments were fused by overlap extension PCR to obtain full-length mutant *gp53* genes. The verified mutant genes were cloned into pET-28a-SUMO for protein expression and pMMB67EH for phenotypic analyses. All constructs were confirmed by DNA sequencing prior to use.

### Biofilm assay

Biofilm formation was evaluated using a modified version of a previously described method^38^. The quantification of biofilm was performed in borosilicate glass tubes. The strains were inoculated into LB medium containing 0.5 mM IPTG at a ratio of 1:100 (v/v) and incubated statically at 25 °C for 12 h. The resulting biofilms were stained with 0.1% crystal violet for 20 min, followed by gentle washing with distilled water to remove unbound dye. After air drying, the retained crystal violet was solubilized with 30% acetic acid, and the absorbance was measured at a wavelength of 560 nm.

### Swarming and swimming assays

Swarming and swimming motility assays were performed as described previously^39^. The swarming medium contained 0.5% agar, 0.8% nutrient broth, and 0.5% glucose, while the swimming medium consisted of 0.3% agar, 1% tryptone and 0.5% sodium chloride. The bacteria cultures was inoculated onto the corresponding plates containing 0.5 mM IPTG. Swarming plates were cultured at 37 °C for 12 h, and swimming plate was cultured at 30 °C for 12 h. The motility patterns were photographed using a Tanon 1600 imaging system.

### RNA-seq analysis

Overnight cultures of *P. aeruginosa* PAO1 pMMB67EH and PAO1 pMM-*gp53* were transferred into fresh LB medium containing 300 μg/mL Cb and 0.5 mM IPTG, and incubated until reaching an OD_600_ of 0.6. Bacterial cells were harvested, rapidly frozen in liquid nitrogen, and sent to Meiji Biomedical Technology Co., Ltd. (Shanghai, China) for total RNA extraction, cDNA library construction, and RNA-seq analysis. RNA sequencing was performed using the Illumina HiSeq^TM^ 2000 platform (San Diego, CA, USA). Differential gene expression analysis was performed using DESeq2. Genes with |log2 fold change | ≥1 and adjusted *P*-value (false discovery rate, FDR) < 0.05 were considered significantly differentially expressed.

### RT-qPCR

*P. aeruginosa* PAO1 pMMB67EH and PAO1 pMM-*gp53* strains were cultured in LB medium containing 300 μg/mL Cb and 0.5 mM IPTG to OD_600_ = 0.6, and bacterial cells were collected by centrifugation. Total RNA was extracted using a bacterial RNA extraction kit (AG, Changsha, China). Then, the Evo M-MLV RT reaction mixture (AG, Changsha, China) was used to synthesize cDNA. RT-qPCR was performed on a BioRad CFX96 Real-Time PCR Detection System (Hercules, California, USA). RT-qPCR reaction volume was 20 μL, containing cDNA template, 10 μL SYBR Green Pro Taq HS Premix (AG, Changsha, China), primers and RNase-free water. Gene expression levels were normalized to *rpsL* as an internal reference, and relative expression was calculated using the 2^−△△Ct^ method. The expression of early-stage genes in phage ΦPA1019 was also examined by RT-qPCR and the late-stage capsid protein-coding gene *gp5* was used as an internal control.

### DNA affinity purification sequencing (DAP-seq) analysis

DAP-seq^40,41^ was performed to identify genomic targets directly bound by Gp53. Sequencing and bioinformatic analyses were carried out by Bluescape Biotechnology (Baoding, China). Raw sequencing reads were quality-filtered using fastp, and high-quality reads were mapped to the *P. aeruginosa* PAO1 reference genome using BWA-MEM. Peak calling was conducted with MACS, and the resulting binding peaks were annotated with ChIPseeker to determine their genomic distribution. Potential Gp53-binding motifs were identified using HOMER, and peak distributions around transcription start sites were visualized to characterize Gp53 binding profiles.

### Protein purification

The *gp53* gene was cloned into the pET28a-SUMO protein expression vector, and the resulting recombinant plasmid pET28a-SUMO-*gp53* was transformed into *E. coli* BL21 (DE3). Overnight cultures were inoculated into fresh LB medium at a 1:100 (v/v) ratio, and protein expression was induced with 0.5 mM IPTG at 16 °C for 20 h with shaking. The harvested cells were disrupted by ultrasonication, and the target protein His-Gp53 was purified using a Ni-NTA affinity column (GE Healthcare) on an AKTA purification system.

### Electrophoretic mobility shift assay (EMSA)

Promoter DNA probes were amplified by PCR. Purified Gp53 protein was incubated with various DNA probes in a 20 μL reaction mixture containing 10 mM Tris-HCl (pH 7.5), 100 mM NaCl, 1 mM DTT, 10% glycerol, and 3 μg/mL salmon sperm DNA. The binding reactions were carried out at 30 °C for 20 min. Samples were separated on a 6% native polyacrylamide gel in 0.5×TBE buffer at 90 V for 90 min. The gel was stained with cel Red for 10 min and visualized using a Tanon 1600 gel imaging system.

### Protein structure prediction and molecular docking

The three-dimensional structure of the Gp53-DNA complex was predicted using bioinformatics tools. The full-length amino acid sequence of Gp53 and the DNA sequence of its target binding region were submitted to the AlphaFold Server (https://alphafoldserver.com/) for structure prediction with default parameters. The predicted protein-DNA complex model was visualized and analyzed using PyMOL. Polar contacts and hydrogen bonds between Gp53 and DNA were identified using PyMOL’s find polar contact function. Key amino acid residues involved in DNA binding were annotated based on hydrogen bond distribution and spatial orientation within the predicted complex structure.

### Laser confocal microscopy

Overnight cultures were diluted 1:100 into fresh LB medium and induced with 0.5 mM IPTG for the indicated time periods. Cells were collected by centrifugation, resuspended in phosphate-buffered saline (PBS), and mounted on glass slides for microscopic observation. Images were captured using an SP8 laser confocal microscope (Leica Microsystems, Germany).

### Statistical analysis

GraphPad Prism 10 was used for statistical analysis. The experiment was repeated at least three times independently. Detailed information about statistical analysis for each experiment was provided in the figure legends. Differences were considered significant when *P* < 0.05 based on Student′s *t*-test or one-way ANOVA.

## Supplementary information

Supplementary Information

Description of Additional Supplementary Files

Supplementary Data 1

Supplementary Data 2

## Data Availability

The RNA-seq, DAP-seq data and genome sequence of phage ΦPA1019 have been deposited in the SRA database of NCBI (PRJNA1363756, PRJNA1363862, PRJNA1368596). Source data for the predicted Gp53 tetramer model used for modeling the Gp53-DNA complex are provided in Supplementary Data 1. The numerical source data underlying the main figures are provided in Supplementary Data 2. Original gel images for Fig. 6 are provided in Supplementary Fig. S12.
