# Supplementary material for: The bacteriophage transcription factor Gp53 suppresses cell division and regulates Pseudomonas aeruginosa physiology
Source: Commun Biol. 2026 Oct 8;9:1275. doi: 10.1038/s42003-026-11031-z (PMC13650114; doi:10.1038/s42003-026-11031-z)
Supplement: Supplementary file 1 [file 42003_2026_11031_MOESM1_ESM.pdf]

Supplementary Information

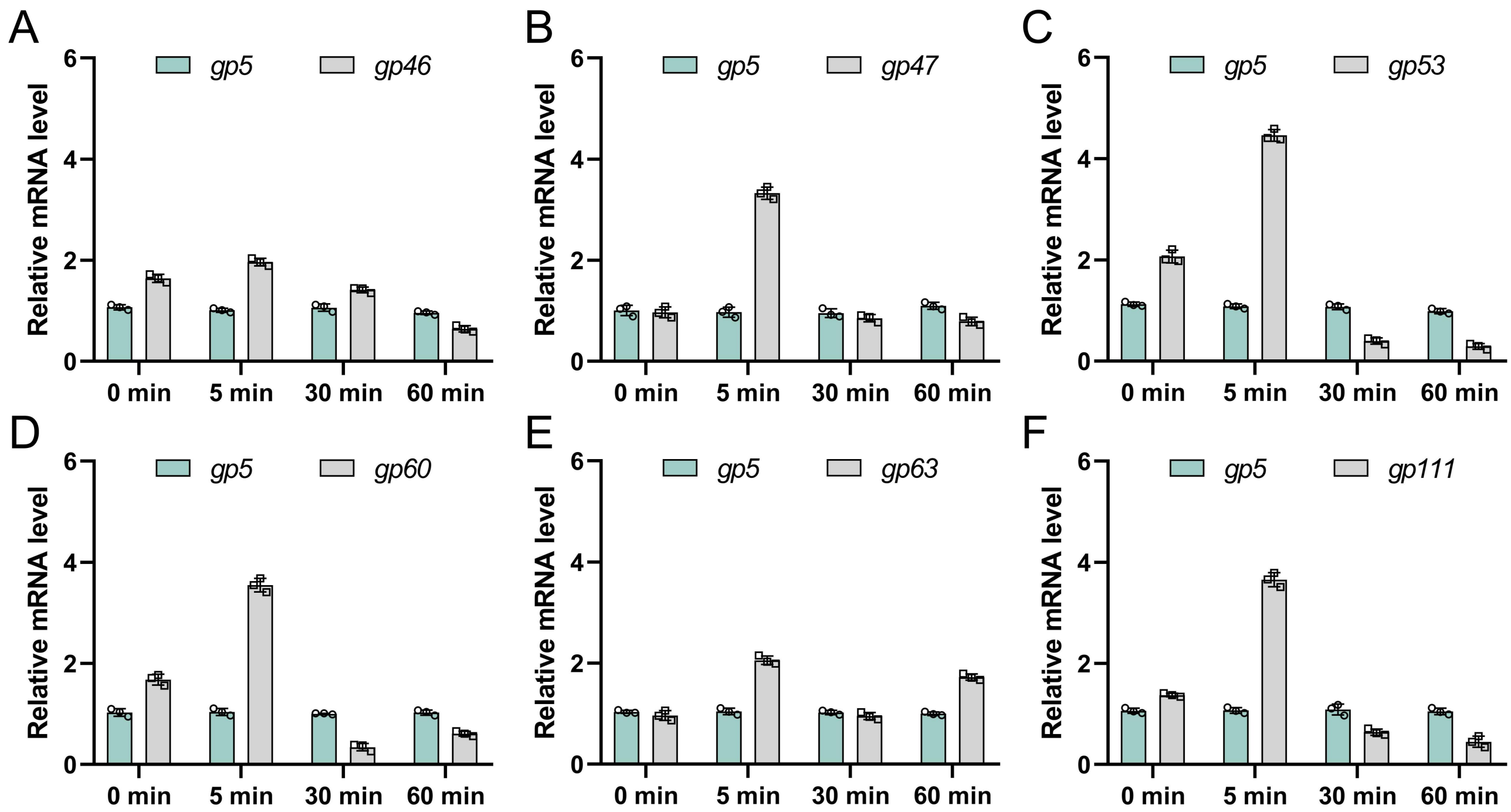

Fig. S1. Expression analysis of early-expressed genes during bacteriophage ΦPA1019 infection. (A–F) RT-qPCR analysis of the transcriptional profiles of ΦPA1019 early-expressed genes *gp46* (A), *gp47* (B), *gp53* (C), *gp60* (D), *gp63* (E), and *gp111* (F) during infection. *P. aeruginosa* PAO1 was inoculated into LB medium at a ratio of 1:100, and cultured at 37℃ for 6 h, followed by infection with ΦPA1019 at a multiplicity of infection (MOI) of 1. Total RNA was extracted from *P. aeruginosa* PAO1 at 0, 5, 30, and 60 minutes post-infection with ΦPA1019, followed by reverse transcription to generate cDNA. The transcriptional profiles of early-stage genes were quantified by RT-qPCR, using the late-stage capsid gene *gp5* as an internal reference. Data are shown as mean ± SD from three biological replicates (n=3).

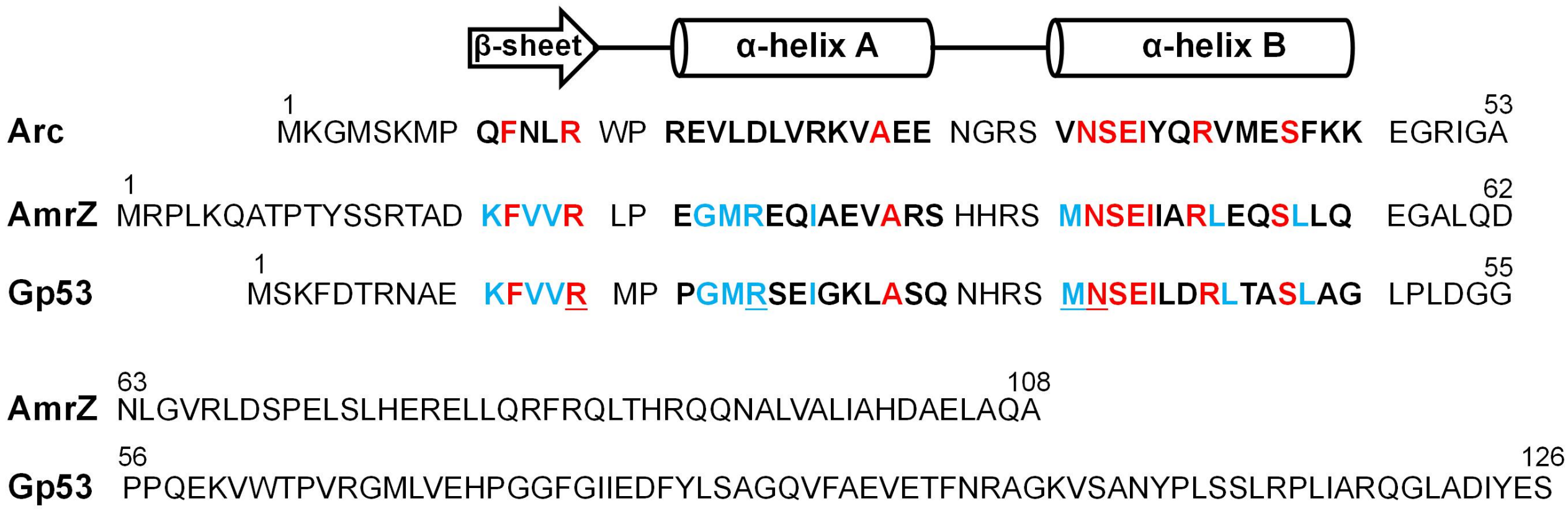

Fig. S2. Protein sequence alignment of Gp53 with Arc and AmrZ. Amino acid residues that correspond to the β-sheet, α-helix A, or α-helix B were indicated in bold. Identical amino acids among Arc (53 aa), AmrZ (108 aa) and Gp53 (126 aa) were shown in red. Other identical amino acids between AmrZ and Gp53 were shown in blue. Numbers at the top indicate the amino acid position. Four amino acids, Arg15, Arg21, Met35 and Asn36 selected for site-directed mutagenesis were underlined.

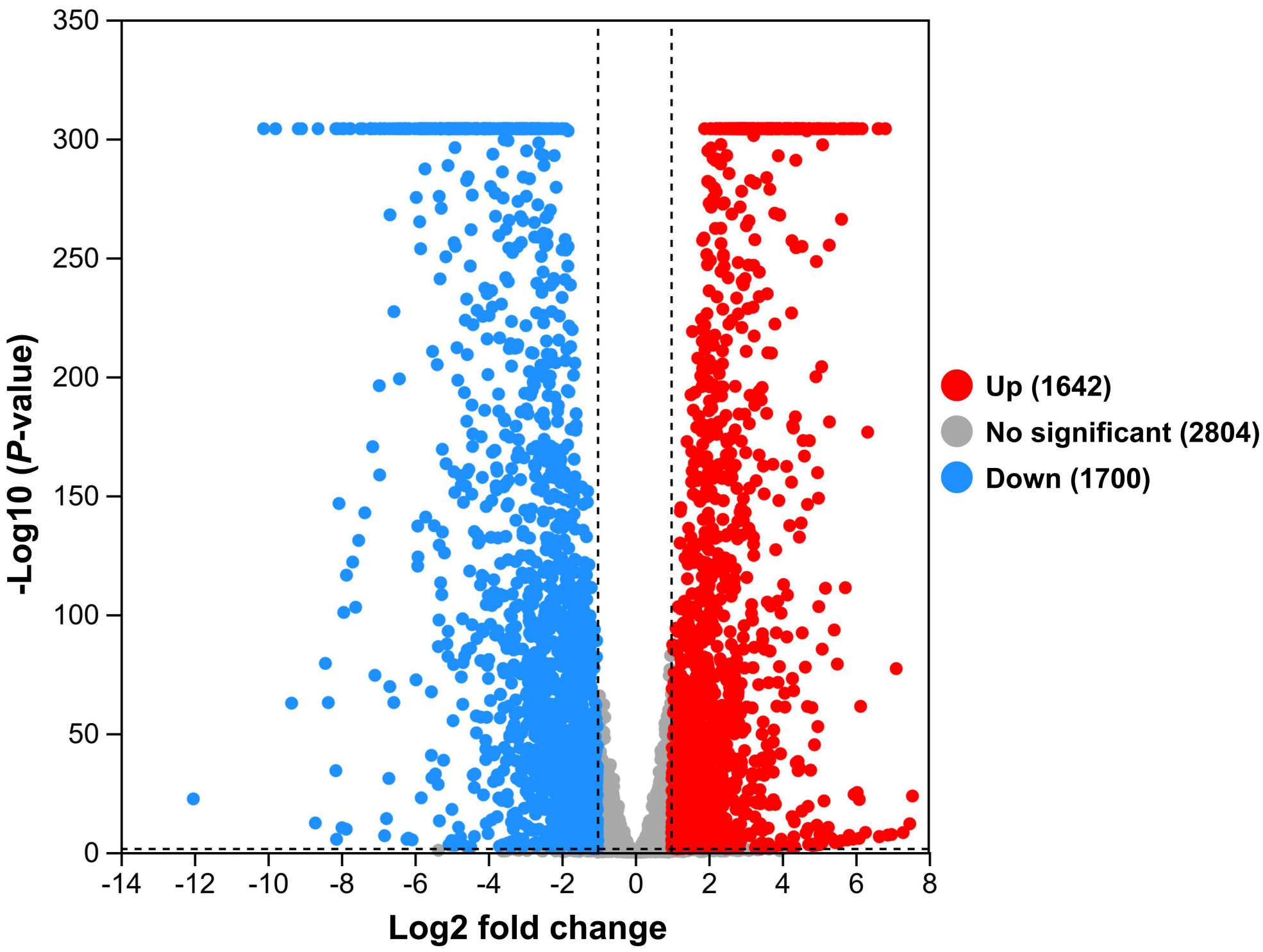

Fig. S3. Volcano plot of RNA-seq data comparing the *gp53*-overexpressing strain with the empty-vector control. Red and blue dots represent significantly upregulated and downregulated genes, respectively, whereas gray dots indicate genes without significant differential expression.

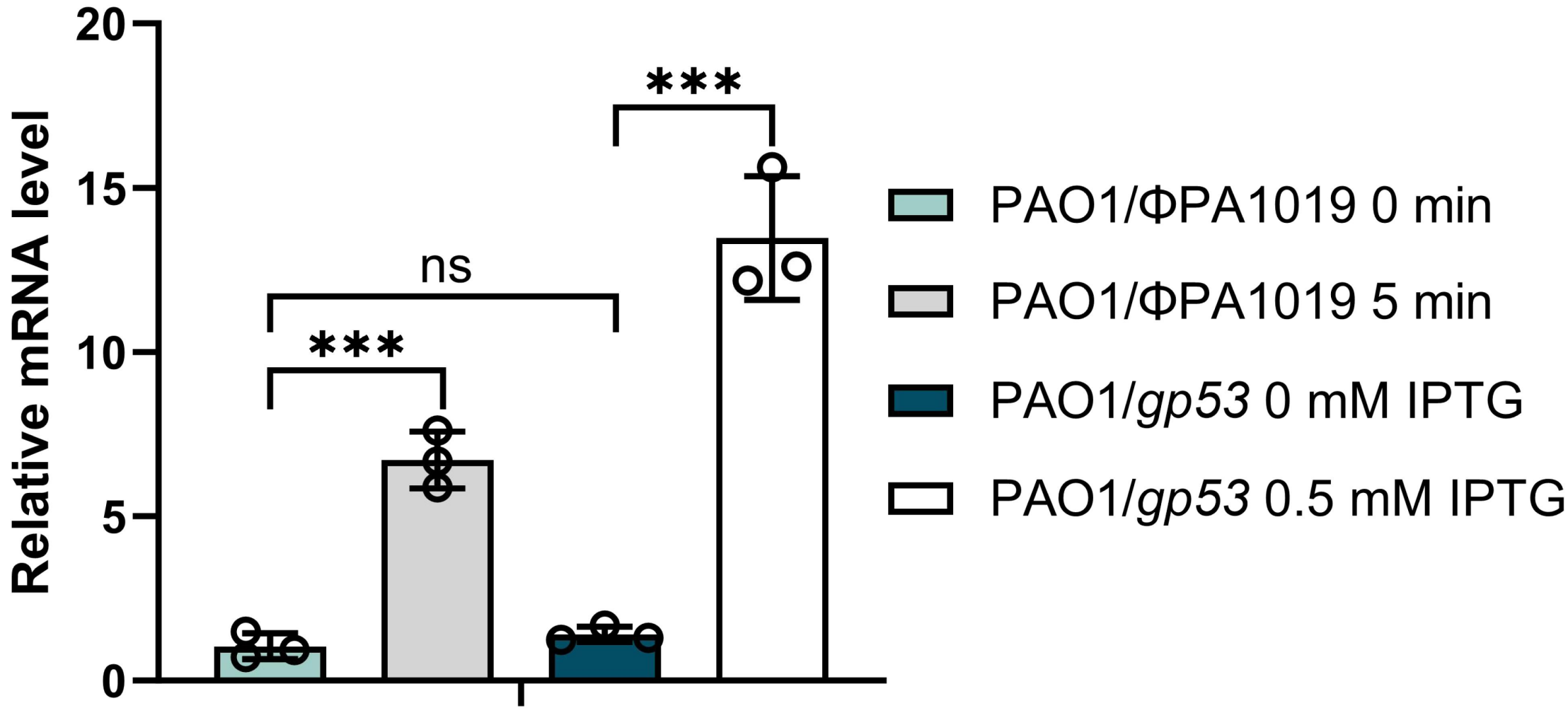

Fig. S4. RT-qPCR analysis of *gp53* expression in *P. aeruginosa* PAO1 under IPTG induction and ΦPA1019 infection. *P. aeruginosa* PAO1 harboring pMM-*gp53* was inoculated into LB medium containing 0 or 0.5 mM IPTG at a ratio of 1:100 and cultured at 37°C for 6 h. For phage infection, *P. aeruginosa* PAO1 was grown in LB medium and cultured at 37℃ for 6 h, followed by infection with ΦPA1019 at a MOI of 1 for 0 and 5 minutes. Samples were collected and total RNA was extracted for subsequent analysis. Expression was normalized to *rpsL*. Data are shown as mean ± SD from three biological replicates (n=3). ****P*<0.001, ns, not significant, based on two-tailed Student′s *t*-test.

TCGCTTTTTCCCGGTTTTATCCGTCCGTCGCAAAAGCGAAAAGCCGCAACTCTTGATA

AATCACTTTAAGTTCGAAGGCTAAGACTTTGGACTTATTGTGTTTTGTCATCTCTTTTAC

TTCTAAAAGCGCTCCGCAACCTTCCAAAAGCACGCTAAATCTCCGTCCTGTAAGGATT

GCANATTGCCATCAT (forward motif1)

TTTCACTTCTGACGCGCCTTGACGGTGGGGAGTGTGGATTTCTATAGTGTGCAGA

-35 box -10 box

AGTGGTATGAAGTGGGTAAAAGTGGGATCAATTGGCATGGAAAGCCAACCACAGG

ATGATGGCAATNTGC (reverse motif1)

GGAAGCGCTGACGTG

Fig. S5. Sequence alignment of the *ftsZ* promoter with the forward and reverse sequences of motif1. The underlined -35 box and -10 box in the promoter region of *ftsZ* were predicted on http://www.softberry.com/. The motif1 consensus sites m1-1 and m1-2 were shown in red and in green.

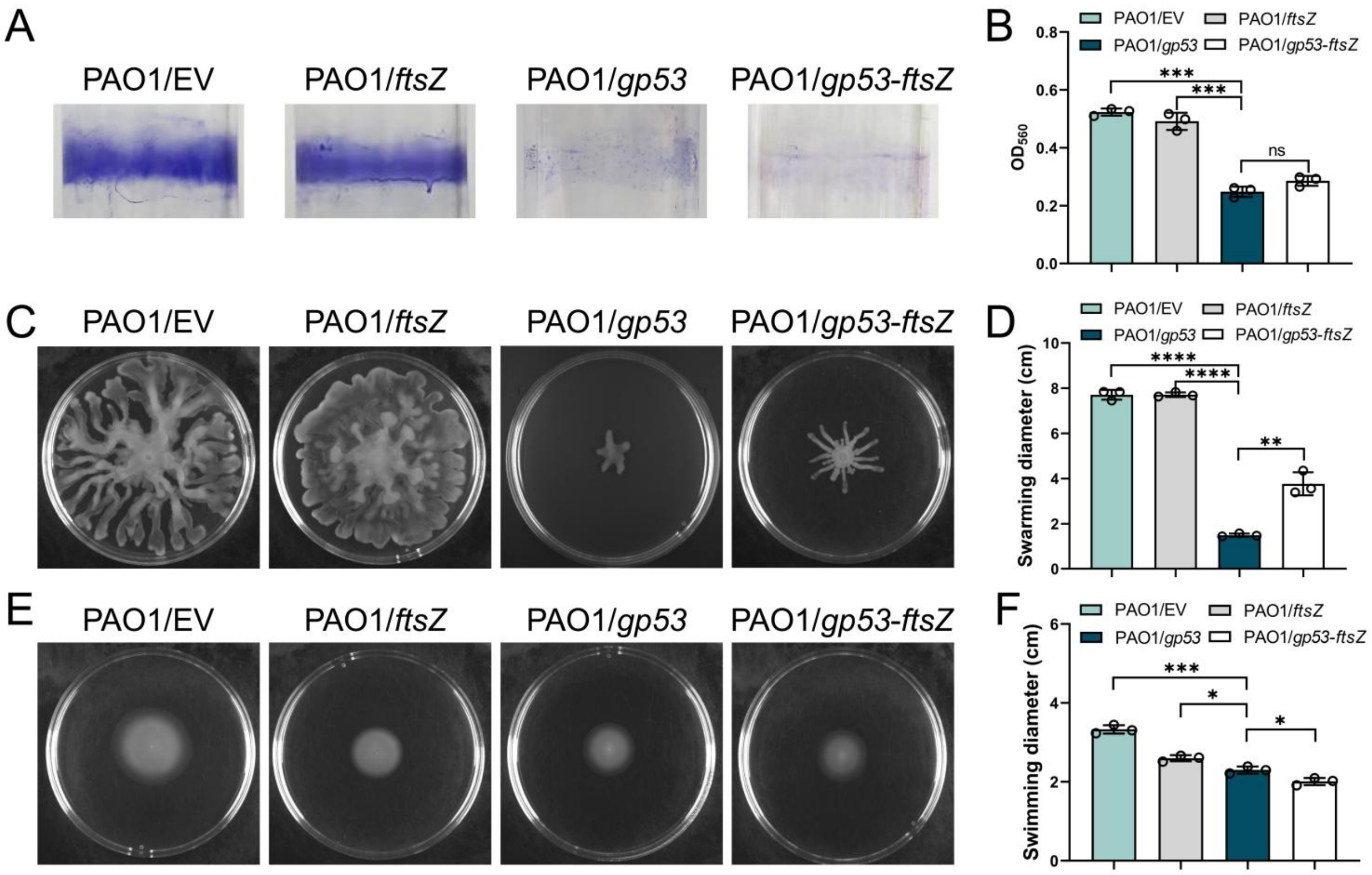

Fig. S6. Co-expression of *ftsZ* and *gp53* does not restore biofilm production and motility in *P. aeruginosa* PAO1. (A) Visualization of biofilm formation. Strains were inoculated into LB medium containing 0.5 mM IPTG at a ratio of 1:100, and cultured at 25℃ for 12 h. Biofilm was stained with 0.1% crystal violet. (B) Quantification of biofilm formation. Stained biofilms were solubilized with 30% acetic acid, and the absorbance was measured at 560 nm. (C) Swarming motility. (E) Swimming motility. Overnight cultures as 2 μL were spotted onto swarming or swimming plates containing 0.5 mM IPTG and incubated at 37℃ or 30℃ for 12 h, respectively. Bacterial motility plates were captured using a Tanon 1600 imaging system. (D) and (F) Measurement of motility zone diameters. Error bars represent the mean ± SD of three independent experiments (n=3). *P<0.05, **P<0.01, ***P<0.001, ****P<0.0001, ns, not significant, determined by two-tailed Student′s *t*-test. PAO1/EV harboring pMMB67EH and pME6032, PAO1/*ftsZ* harboring pMMB67EH and pME-*ftsZ*, PAO1/*gp53* harboring pMM-*gp53* and pME6032, PAO1/*gp53-ftsZ* harboring pMM-*gp53* and pME-*ftsZ*.

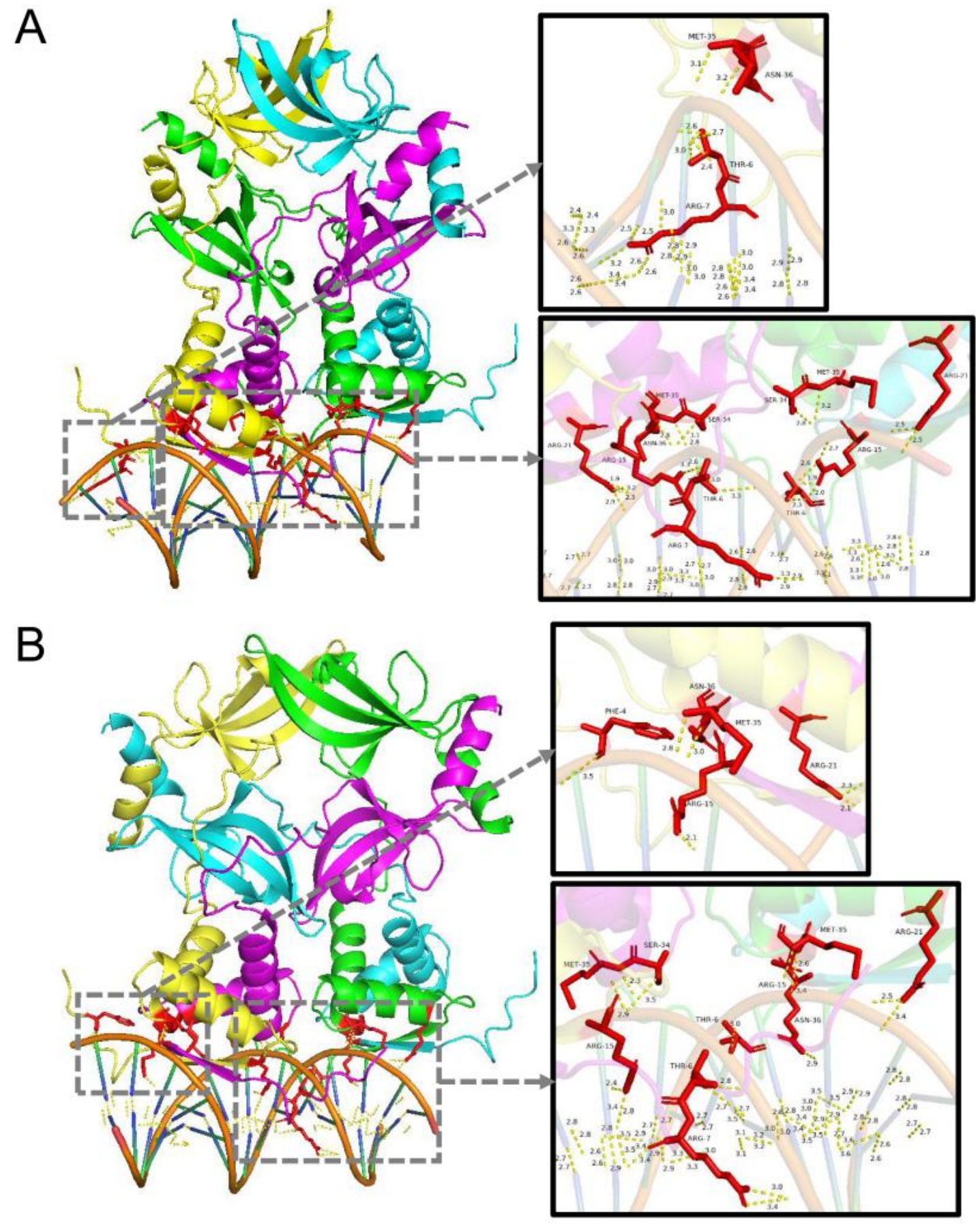

Fig. S7. Molecular docking of Gp53 with *ftsZ* promoter regions m1-1 (A) and m1-2 (B). The complex structure of the Gp53 tetramer and *ftsZ* m1-1 and m1-2 promoter DNA sequences was predicted using the AlphaFold Server, and visual analysis was performed using PyMOL software.

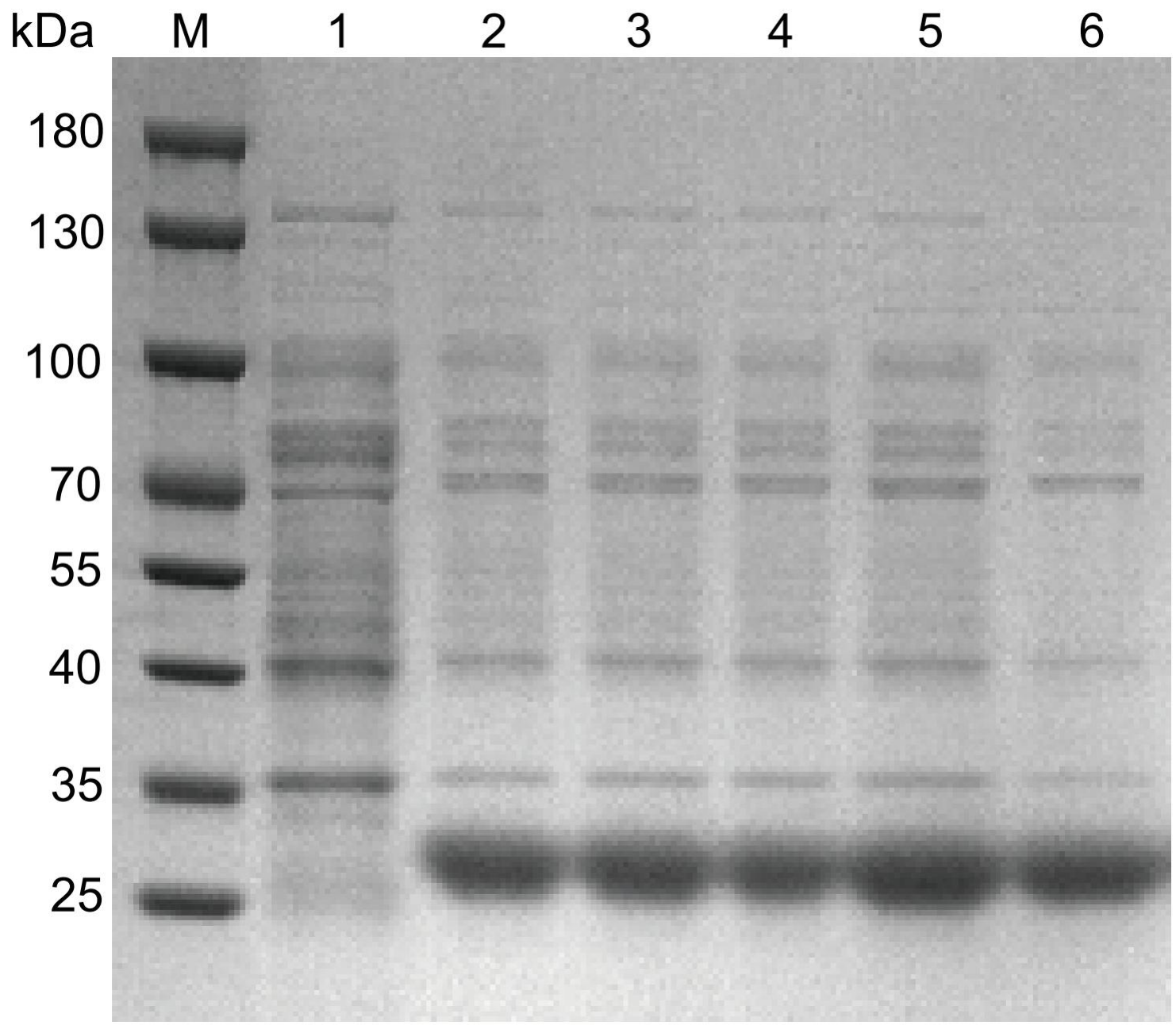

Fig. S8. Expression of Gp53 site-directed mutant proteins. Overnight cultures were inoculated into fresh LB medium containing 0.5 mM IPTG at a ratio of 1:100 (v/v), and incubated with shaking at 16°C for 20 h. Protein samples were collected and analyzed by 12% SDS–PAGE. M: 10-180 kDa protein marker; lane 1: total protein of BL21(DE3) pET28a-SUMO cells (negative control); lane 2: total protein of BL21(DE3) cells expressing wild-type Gp53 (positive control); lane 3-6: total proteins of BL21(DE3) cells expressing the site-directed mutants $Gp53^{R15A}$, $Gp53^{R21A}$, $Gp53^{M35A}$ and $Gp53^{N36A}$.

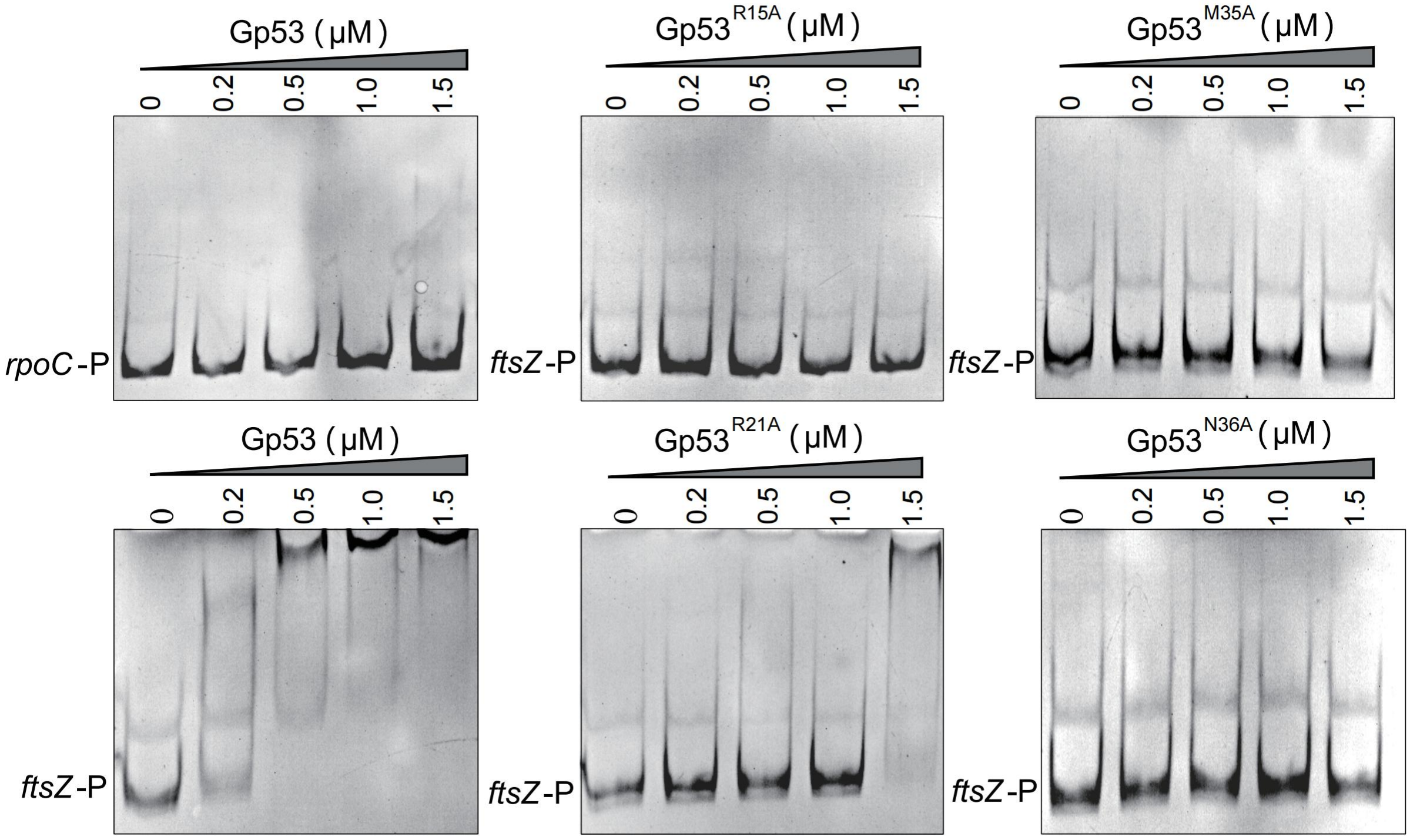

Fig. S9. EMSA analysis of Gp53 mutants binding to the *ftsZ* promoter. Purified Gp53 mutant proteins were incubated with 50 ng of *ftsZ* promoter DNA in 20 μL binding buffer. Gp53 mutant proteins were added to lanes 2-5, while lane 1 served as a negative control with no protein.

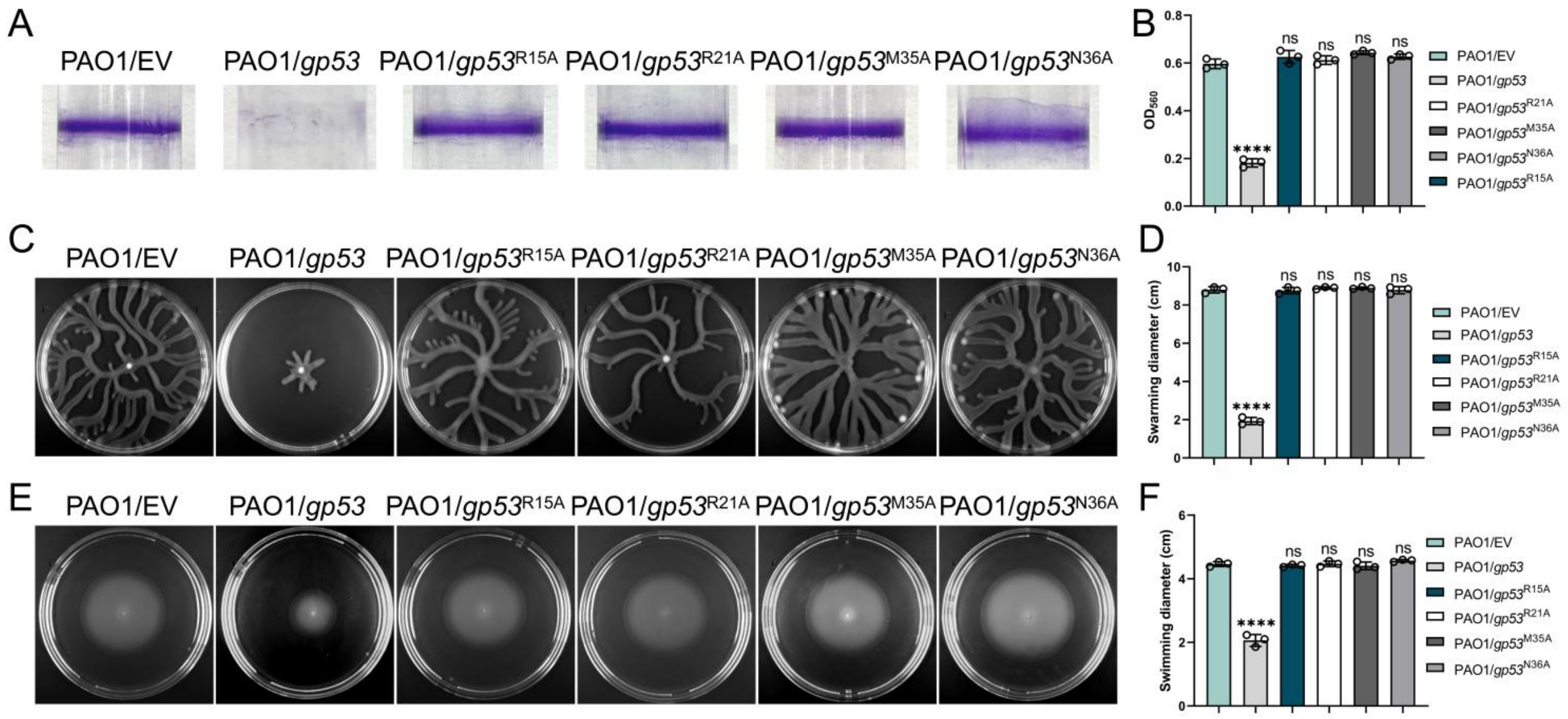

Fig. S10. Effects of Gp53 mutations on biofilm formation and motility in *P. aeruginosa* PAO1. (A) Visualization of biofilm formation. Strains were inoculated into LB medium containing 0.5 mM IPTG at a ratio of 1:100, and cultured at 25℃ for 12 h. Biofilm was stained with 0.1% crystal violet. (B) Quantification of biofilm formation. Stained biofilms were solubilized with 30% acetic acid, and the absorbance was measured at 560 nm. (C) Swarming motility. (E) Swimming motility. Overnight cultures as 2 μL were spotted onto swarming or swimming plates containing 0.5 mM IPTG and incubated at 37℃ or 30℃ for 12 h, respectively. Bacterial motility plates were captured using a Tanon 1600 imaging system. (D) and (F) Measurement of motility zone diameters. Error bars represent the mean ± SD of three independent experiments (n=3). Statistical significance was determined relative to the PAO1/EV control group. *****P*<0.0001, ns, not significant, determined by two-tailed Student′s *t*-test.

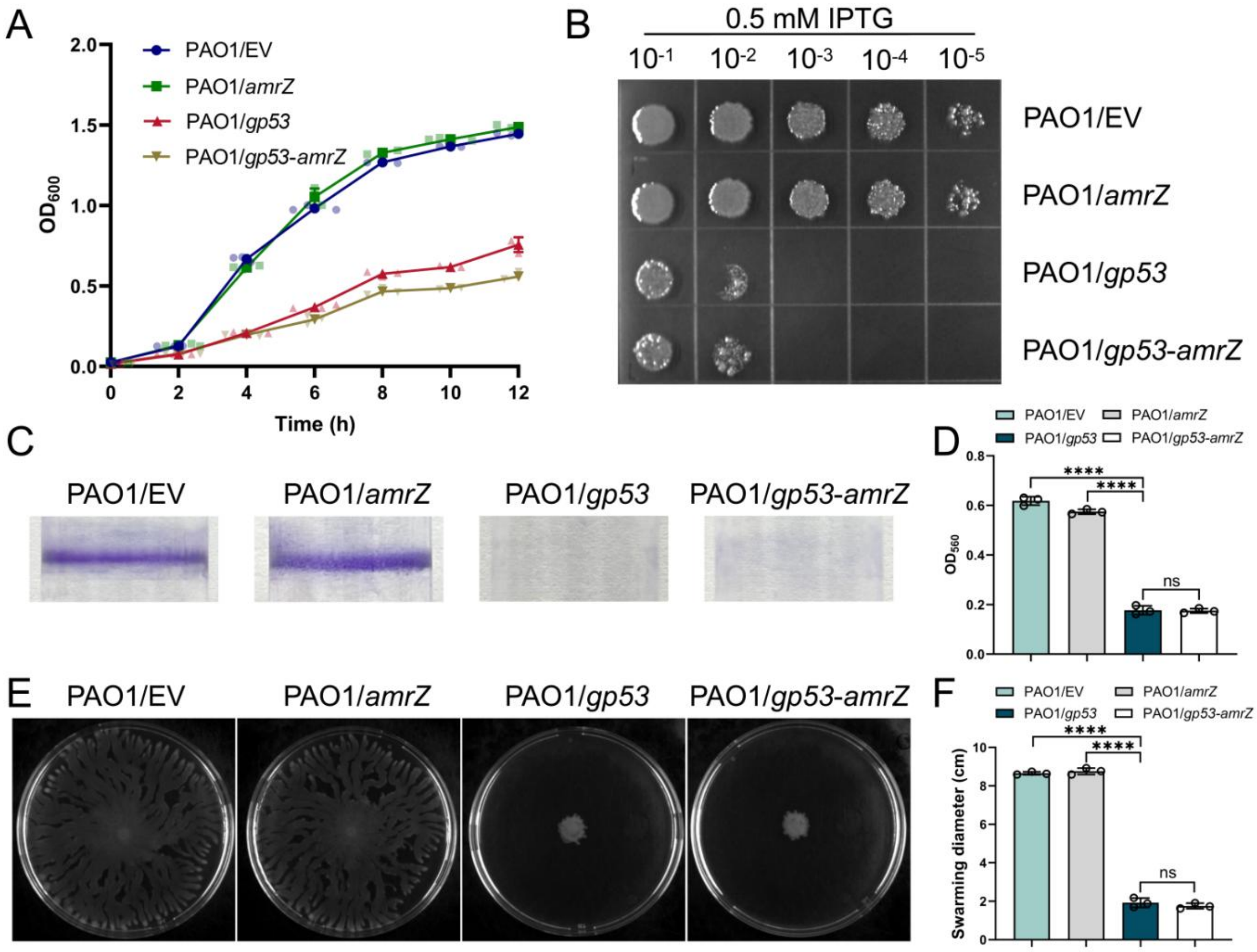

Fig. S11. Co-expression of *amrZ* and *gp53* does not restore bacterial growth, biofilm production and motility in *P. aeruginosa* PAO1. (A) Bacterial growth curves. Strains were inoculated into LB medium containing 0.5 mM IPTG and cultured with shaking at 37℃. Optical density at 600 nm was measured every 2 h. Data represent mean ± SD from three biological replicates (n=3). (B) Agar spot assay for growth assessment. Cultures grown for 6 h were serially diluted and spotted onto LB agar plates, followed by incubation at 37℃ for 24 h. (C) Visualization of biofilm formation. Strains were inoculated into LB medium containing 0.5 mM IPTG at a ratio of 1:100, and cultured at 25℃ for 12 h. Biofilm was stained with 0.1% crystal violet. (D) Quantification of biofilm formation. Stained biofilms were solubilized with 30% acetic acid, and the absorbance was measured at 560 nm. (E) Swarming motility. Overnight cultures as 2 μL were spotted onto swarming plates containing 0.5 mM IPTG and incubated at 37℃ for 12 h, respectively. Bacterial motility plates were captured using a Tanon 1600 imaging system. (F) Measurement of motility zone diameters. Error bars represent the mean ± SD of three independent experiments (n=3). ****P<0.0001, ns, not significant, determined by two-tailed Student′s t-test.

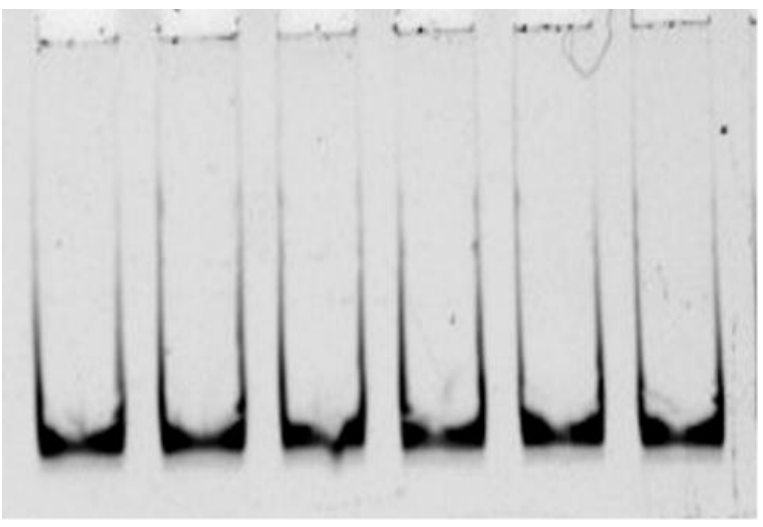

Figure 6A

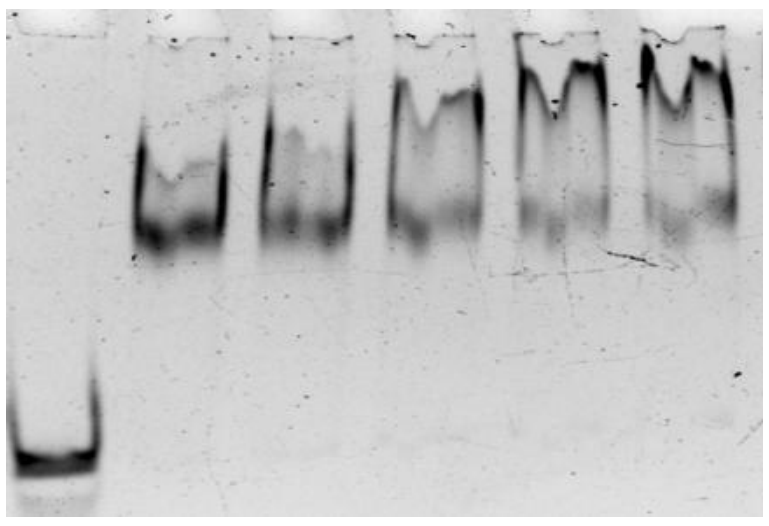

Figure 6B

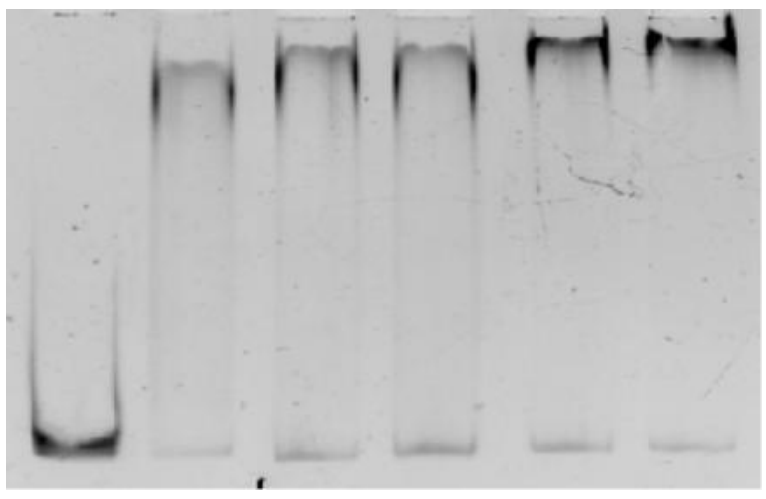

Figure 6C

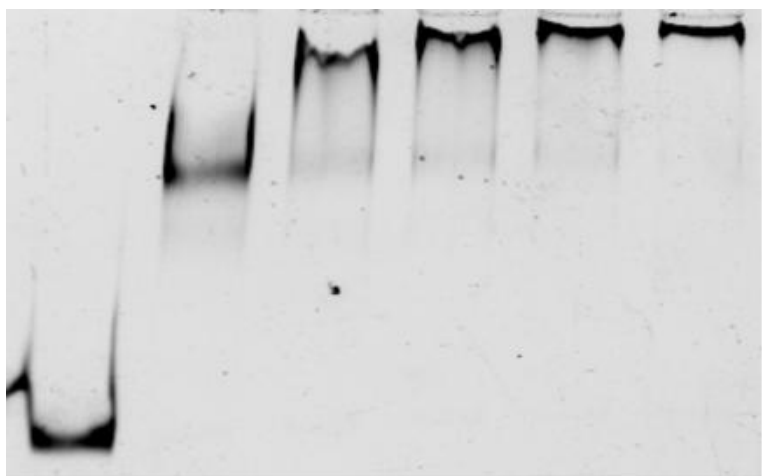

Figure 6D

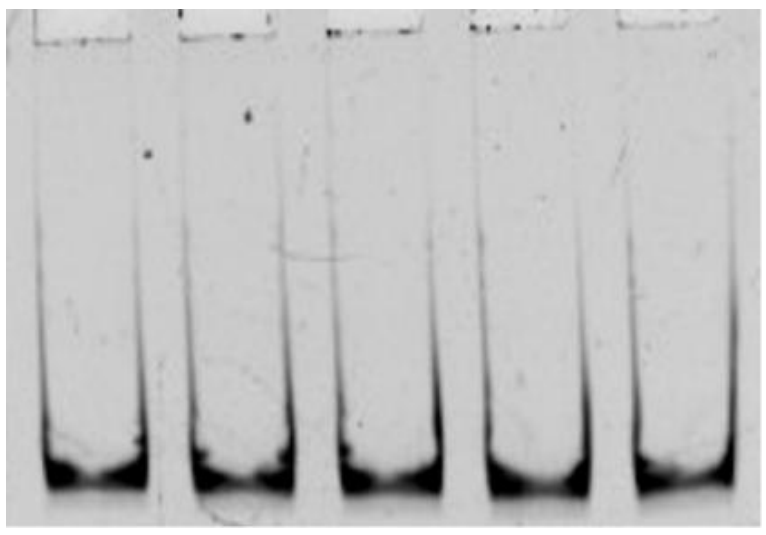

Figure 6E

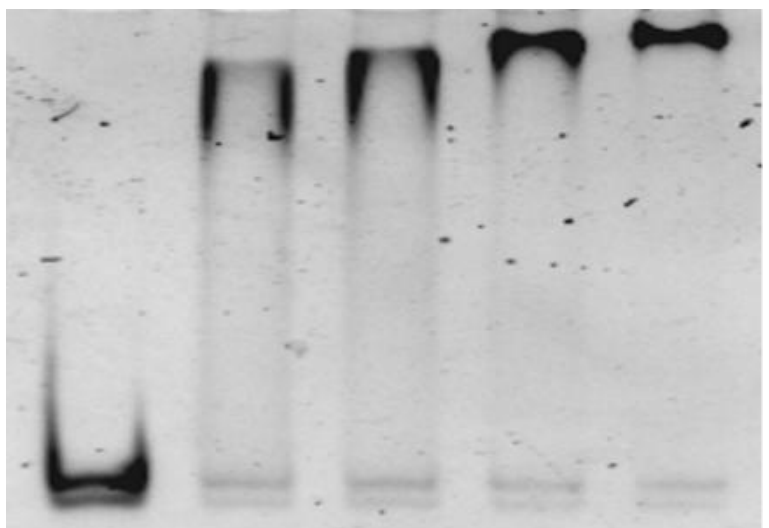

Figure 6F

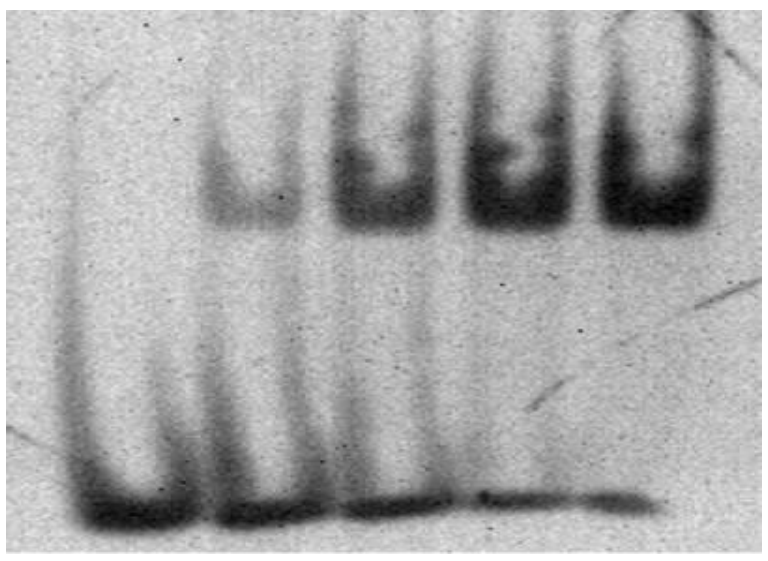

Figure 6G

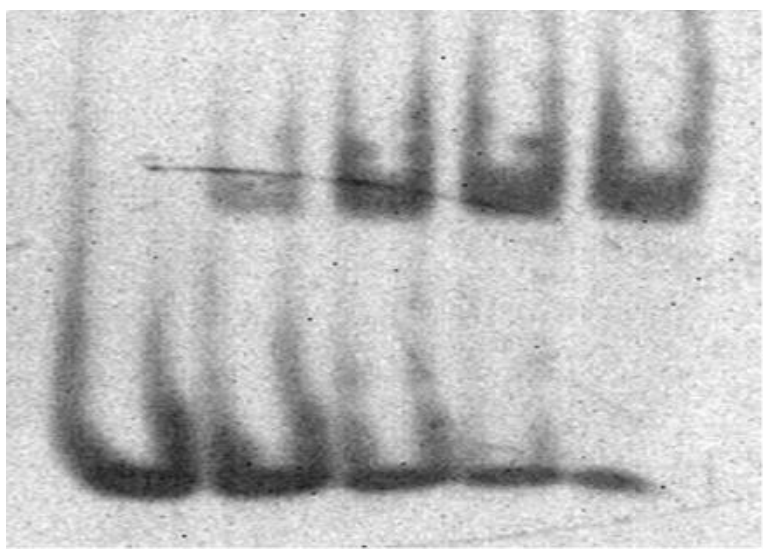

Figure 6H

Fig. S12. Original gel images for Figure 6.

Table S1. Strains and plasmids used in this study.

Strains or plasmids	Relevant characteristics	Source
E. coli		
DH5α	*F– φ80lacZ ΔM15 Δ(lacZYA-argF)U169 recA1 endA1 hsdR17(rk–, mk+) phoA supE44 thi-1 gyrA96 relA1 tonA*	Sangon
BL21(DE3)	F^{-} *ompT gal dcm lon hsdS*$_{B}$(r_{B}^{-} m_{B}^{-}) λ(DE3)	Sangon
P. aeruginosa		
PAO1	Wild type	This lab
**Plasmids**		
pMMB67EH	*E. coli-P. aeruginosa* shuttle cloning vector; Cb^{r}	1
pMM-*gp53*	Insert the entire *gp53* gene into pMMB67EH; Cb^{r}	This study
pMM-*gp63*	Insert the entire *gp63* gene into pMMB67EH; Cb^{r}	This study
pMM-*gp53*^{R15A}	Insert the entire *gp53*^{R15A} gene into pMMB67EH; Cb^{r}	This study
pMM-*gp53*^{R21A}	Insert the entire *gp53*^{R21A} gene into pMMB67EH; Cb^{r}	This study
pMM-*gp53*^{M35A}	Insert the entire *gp53*^{M35A} gene into pMMB67EH; Cb^{r}	This study
pMM-*gp53*^{N36A}	Insert the entire *gp53*^{N36A} gene into pMMB67EH; Cb^{r}	This study
pME6032	*E. coli-P. aeruginosa* shuttle cloning vector; Tc^{r}	2
pME-*zapA*-GFP	Insert the entire *zapA* gene fused with GFP into pME6032; Tc^{r}	This lab
pME-*ftsZ*	Insert the entire *ftsZ* gene into pME6032; Tc^{r}	This study
pME-*amrZ*	Insert the entire *amrZ* gene into pME6032; Tc^{r}	This study
pMM-*gp53-ftsZ*	Insert the entire *gp53* gene and *ftsZ* gene into pMMB67EH; Cb^{r}	This study
pET-28a-SUMO	Protein expression vector with His and SUMO tag; Kn^{r}	This lab
pET-28a-SUMO-*gp53*	Insert the entire *gp53* gene into protein expression vector pET-28a-SUMO; Kn^{r}	This study
pET-28a-SUMO-*gp53*^{R15A}	Insert the entire *gp53*^{R15A} gene into protein expression vector pET-28a-SUMO; Kn^{r}	This study
pET-28a-SUMO-*gp53*^{R21A}	Insert the entire *gp53*^{R21A} gene into protein expression vector pET-28a-SUMO; Kn^{r}	This study
pET-28a-SUMO-*gp53*^{M35A}	Insert the entire *gp53*^{M35A} gene into protein expression vector pET-28a-SUMO; Kn^{r}	This study
pET-28a-SUMO-*gp53*^{N36A}	Insert the entire *gp53*^{N36A} gene into protein expression	This study

vector pET-28a-SUMO; Kn^r

References

1. Fürste J P, Pansegrau W, Frank R, et al. Molecular cloning of the plasmid RP4 primase region in a multi-host-range *tacP* expression vector [J]. Gene, 1986, 48(1): 119-131
2. Heeb S, Itoh Y, Nishijyo T, et al. Small, stable shuttle vectors based on the minimal pVS1 replicon for use in gram-negative, plant-associated bacteria [J]. Molecular plant-microbe interactions, 2000, 13(2): 232-237

Table S2. Primers used in this study.

Primer	Sequence(5'→3')	Application
pMM-*gp53*-S	CTGgaattcATGAGCAAATTCGACACG	For constructing pMM-*gp53*
pMM-*gp53*-AS	ACGtctagaTCAGGACTCGTAGATGTC	
pMM-*gp63*-S	TGCgaattcATGACCATTGCAAAAGAT	For constructing pMM-*gp63*
pMM-*gp63*-AS	ACGtctagaCTATCTCAGGATATCTCG	
pME-*ftsZ*-S	CGCgaattcATGTTTGAACTGGTCGATAAC	For constructing pME-*ftsZ*
pME-*ftsZ*-AS	ATActcgagTCAATCGGCCTGACGACG	
pME-*amrZ*-S	ATAgaattcATGCGCCCACTGAAACAG	For constructing pME-*amrZ*
pME-*amrZ*-AS	ATAggtaccTCAGGCCTGGGCCAGCTC	
pMM-*ftsZ*-S	CCGtctagaTTCAACGCGGTAACAGTA	For constructing pMM-*gp53*-*ftsZ*
pMM-*ftsZ*-AS	ATAtctagaTCAATCGGCCTGACGACG	
pMM-*gp53*^{R15A}-S	GAAGTTCGTGGTCGCTATGCCGCCTGGGA	For constructing pMM-*gp53*^{R15A}
pMM-*gp53*^{R15A}-AS	TCCCAGGCGGCATAGCGACCACGAACTTC	
pMM-*gp53*^{R21A}-S	GCCGCCTGGGATGGCCTCCGAGATCGGTA	For constructing pMM-*gp53*^{R21A}
pMM-*gp53*^{R21A}-AS	TACCGATCTCGGAGGCCATCCCAGGCGGC	
pMM-*gp53*^{M35A}-S	AAATCACCGCAGCGCTAACTCCGAAATCC	For constructing pMM-*gp53*^{R35A}
pMM-*gp53*^{M35A}-AS	GGATTTCGGAGTTAGCGCTGCGGTGATTT	
pMM-*gp53*^{N36A}-S	TCACCGCAGCATGGCCTCCGAAATCCTCG	For constructing pMM-*gp53*^{R36A}
pMM-*gp53*^{N36A}-AS	CGAGGATTTCGGAGGCCATGCTGCGGTGA	
pET-28a-*gp53*-S	TGTgaattcATGAGCAAATTCGACACGC	For constructing pET-28a-SUMO-*gp53*
pET-28a-*gp53*-AS	ACCaagcttTCAGGACTCGTAGATGTCGG	
RT-qPCR-*gp5*-S	AGCGCGTCTAAGCTTCAAGT	For RT-qPCR
RT-qPCR-*gp5*-AS	TACACTACCGGGCACTACCA	
RT-qPCR-*gp46*-S	CTGTGCAGACTTGGACCTGT	For RT-qPCR
RT-qPCR-*gp46*-AS	TTTCTTTCGCGGGTCGTACT	
RT-qPCR-*gp47*-S	CTCGGGTATTCCAGTGGCTC	For RT-qPCR
RT-qPCR-*gp47*-AS	TGGGCCCGAAAGACCTAGTA	
RT-qPCR-*gp48*-S	GATACTCCGGCTTCCGAGTG	For RT-qPCR
RT-qPCR-*gp48*-AS	TCGAAACTATCGGTCTGCCG	
RT-qPCR-*gp53*-S	CCGGGCAAGTTTTTGCTGAA	For RT-qPCR
RT-qPCR-*gp53*-AS	CAGGACTCGTAGATGTCGGC	
RT-qPCR-*gp59*-S	TTCGTCTGGAGAACGCGAAA	For RT-qPCR
RT-qPCR-*gp59*-AS	ACATGTGGTGGCTAGGCTTC	
RT-qPCR-*gp60*-S	CATTGATTTGGCTACCGGCG	For RT-qPCR
RT-qPCR-*gp60*-AS	AATTGTGCTCTGCGCTCTCT	

RT-qPCR-*gp62*-S	TGTACGAGGAGGTAGCGGAA	For RT-qPCR
RT-qPCR-*gp62*-AS	AGTCCCCCACAACTGCAAAA	
RT-qPCR-*gp63*-S	TTCCCCTTTGCCTACGTCAC	For RT-qPCR
RT-qPCR-*gp63*-AS	CGGGTGCCTTTGTACTCACT	
RT-qPCR-*gp92*-S	CGCGAGTTCGACATCGTTTC	For RT-qPCR
RT-qPCR-*gp92*-AS	CCGGAGAATACCGTCAGAGC	
RT-qPCR-*gp111*-S	TTACGGCATCGAAAAGCCCT	For RT-qPCR
RT-qPCR-*gp111*-AS	GGCAAGGGAGAGTTACGGAG	
RT-qPCR-*gp112*-S	CCGCAGATACTGGCGAAGAT	For RT-qPCR
RT-qPCR-*gp112*-AS	GGGGAGTAAGCGTACCGATG	
RT-qPCR-*gp117*-S	GGTGACGACGATGAAACCCT	For RT-qPCR
RT-qPCR-*gp117*-AS	TTCGAGGAATTCCGGGTTGG	
RT-qPCR-*rpsL*-S	GTAAGGTATGCCGTGTACG	For RT-qPCR
RT-qPCR-*rpsL*-AS	CACTACGCTGTGCTCTTG	
RT-qPCR-*sulA*-S	CGCTGCCTTTAGCGAACTTT	For RT-qPCR
RT-qPCR-*sulA*-AS	CCATTCATGGGTCAGGCTCG	
RT-qPCR-*minC*-S	GGTAACTACAAGGTCGCGGA	For RT-qPCR
RT-qPCR-*minC*-AS	ATGTTCAACACGTCACCCGA	
RT-qPCR-*minD*-S	GTGATCCTCGACGAGCAGAG	For RT-qPCR
RT-qPCR-*minD*-AS	ATGCGGTATTTCCTTGCCGA	
RT-qPCR-*zapA*-S	CCAGTCGAATACCCTCACCG	For RT-qPCR
RT-qPCR-*zapA*-AS	ATCTTGCCGTCCAGGTAACG	
RT-qPCR-*ftsZ*-S	AGAAACCGGTGAAGGTCGTC	For RT-qPCR
RT-qPCR-*ftsZ*-AS	GTAGTTCACCGACTGCTGCT	
RT-qPCR-*ftsA*-S	ATCAAGATCCGCTACGCCTG	For RT-qPCR
RT-qPCR-*ftsA*-AS	GGGTGAACAGCTCGTCGTAA	
RT-qPCR-*amrZ*-S	CTCCTACCTACTCCAGCCGT	For RT-qPCR
RT-qPCR-*amrZ*-AS	CGACTTCTGCGATCTGCTCA	
RT-qPCR-*fleQ*-S	GATGATCGAGGACGGCACTT	For RT-qPCR
RT-qPCR-*fleQ*-AS	TCCATCCGCGAGATCAGTTC	
RT-qPCR-*pslB*-S	GCGCTGTTCCTGCTGTACTA	For RT-qPCR
RT-qPCR-*pslB*-AS	CAGGCGAAGAACATGATGCG	
RT-qPCR-*flgA*-S	TTTACCGCTCCCGAGGTACT	For RT-qPCR
RT-qPCR-*flgA*-AS	CTGAGCGTCAAGTCCCTGTC	
amrZ-promoter-S	CGTTCTCCCTCCAATCGGTT	*amrZ* promoter
amrZ-promoter-AS	ACATTGAACCTGTAGAGTCAGGG	

fleQ-promoter-S	GTTTTTCATGGCTTTGTGCC	*fleQ* promoter
fleQ-promoter-AS	TTTGATCAGCTGCCTTGCATC	
pslA-promoter-S	AGCCGTCCGCGCACGCCG	*pslA* promoter
pslA-promoter-AS	GTTGTTTGCTCTGCCGATCACG	
ftsZ-promoter-S	GTCAGCGCTTCCCCTGTG	*ftsZ* promoter
ftsZ-promoter-AS	TCGCTTTTTCCCGGTTTTAT	
minC-promoter-S	GGACGGTTCCGACGACGA	*minC* promoter
minC-promoter-S	GGGGCTTGTCGGTCGTTG	
rpoC-promoter-S	CAGCGTTCCCCAGCTTGG	*rpoC* promoter
rpoC-promoter-S	TTTTCCGTTCTGGGTGGACTC	
ftsZ-m1-1-S	GTGGATTTCTATAGT	*ftsZ* promoter m1-1
ftsZ-m1-1-AS	ACTATAGAAATCCAC	
ftsZ-m1-2-S	GCCAATTGATCCCAC	*ftsZ* promoter m1-2
ftsZ-m1-2-AS	GTGGGATCAATTGGC	

Table S3. Lytic spectrum of bacteriophage ΦPA1019.

***P. aeruginosa* strain**	**Source**	**Lytic property**
PAO1	This lab	+
PA10-15	This lab	+
PA10-23	This lab	+
PA10-24	This lab	+
PA14	This lab	–
PAYQ48	This lab	–
PAYQ64	This lab	–
PAYQ66	This lab	–
PAYQ78	This lab	–
PA1	Nanjing public health medical centre	+
PA2	Nanjing public health medical centre	+
PA13	Nanjing public health medical centre	+
PA15	Nanjing public health medical centre	–
PA16	Nanjing public health medical centre	+
PA19	Nanjing public health medical centre	+
PA21	Nanjing public health medical centre	+
PA22	Nanjing public health medical centre	+
PA25	Nanjing public health medical centre	–
PA31	Nanjing public health medical centre	–
PA33	Nanjing public health medical centre	+
PA34	Nanjing public health medical centre	+
PA36	Nanjing public health medical centre	+
PA37	Nanjing public health medical centre	+
PA39	Nanjing public health medical centre	+
PA40	Nanjing public health medical centre	+
PA41	Nanjing public health medical centre	+
PA42	Nanjing public health medical centre	+
PA49	Nanjing public health medical centre	–
PA51	Nanjing public health medical centre	+
PA63	Nanjing public health medical centre	–

Table S4. Functional annotation of putative early-expressed genes in bacteriophage ΦPA1019.

Gene ID	Nucleotide position	Putative function
gp46	22118-22354	putative DNA methylase
gp47	22320-22628	MazF family toxin-antitoxin system protein
gp48	22666-23460	lysozyme
gp53	25134-25514	Arc-like transcriptional regulator
gp59	28229-29380	DNA polymerase III alpha subunit
gp60	29340-30032	DNA-binding protein
gp62	30789-31442	ATP-dependent helicase
gp63	31439-32185	MazG-like pyrophosphatase
gp92	60223-61947	putative AAA+ ATPase
gp111	76873-77226	putative transcriptional regulator
gp112	77229-78431	putative RecA protein
gp117	80716-82053	putative single-stranded DNA binding protein

Table S5. Differentially expressed genes involved in cell division and pathogenicity.

Gene ID	Gene name	Description	Log2 fold change
**Genes related to cell division**			
PA3008	*sulA*	cell division inhibitor SulA	3.111
PA3243	*minC*	cell division inhibitor MinC	3.028
PA3244	*minD*	cell division inhibitor MinD	1.913
PA3245	*minE*	cell division topological specificity factor MinE	1.874
PA4407	*ftsZ*	cell division protein FtsZ	-1.792
PA4408	*ftsA*	cell division protein FtsA	-1.633
PA5227	*zapA*	cell division protein ZapA	-1.311
**Genes related to quorum sensing system**			
PA1430	*lasR*	transcriptional regulator LasR	-2.565
PA1432	*lasI*	autoinducer synthesis protein LasI	-1.163
PA3476	*rhlI*	autoinducer synthesis protein RhlI	-5.606
PA3477	*rhlR*	transcriptional regulator RhlR	-4.833
PA1003	*mvfR/pqsR*	transcriptional regulator MvfR	-2.070
PA2587	*pqsH*	FAD-dependent monooxygenase	-3.513
**Genes related to type III secretion system**			
PA1710	*exsC*	T3SS regulatory chaperone ExsC	-3.177
PA1711	*exsE*	T3SS regulon translocated regulator ExsE	-2.258
PA1712	*exsB*	exoenzyme S synthesis protein B	-1.254
**Genes related to type VI secretion system**			
PA0085	*hcp1*	type VI secretion protein Hcp1	-1.561
PA1511	*vgrG2*	type VI secretion protein VgrG2	-2.891
PA1512	*hcpA/hcp2*	type VI secretion protein HcpA	-7.076
PA2367	*hcp3*	type VI secretion protein Hcp3	-3.405
PA2373	*vgrG3*	type VI secretion protein VgrG3	-3.518
**Genes related to pyocyanin**			
PA4210	*phzA1*	phenazine biosynthesis protein PhzA1	-5.331
PA4211	*phzB1*	phenazine biosynthesis protein PhzB1	-8.622
PA1899	*phzA2*	phenazine biosynthesis protein PhzA2	-2.355
PA1900	*phzB2*	phenazine biosynthesis protein PhzB2	-3.837
**Genes related to rhamnolipid**			
PA3478	*rhlB*	rhamnosyltransferase chain B	-3.877

PA3479	*rhlA*	rhamnosyltransferase chain A	-2.537
PA1130	*rhlC*	rhamnosyltransferase 2	-3.298
PA5161	*rmlB*	dTDP-D-glucose 4,6-dehydratase	-2.018
PA5162	*rmlD*	dTDP-4-dehydrorhamnose reductase	-1.834
PA5163	*rmlA*	glucose-1-phosphate thymidylyltransferase	-2.488
PA5164	*rmlC*	dTDP-4-dehydrorhamnose 3,5-epimerase	-2.993
**Genes related to other virulence proteins**			
PA1148	*toxA*	exotoxin A	-1.108
PA1249	*aprA*	alkaline metalloproteinase	-6.792
PA1871	*lasA*	protease LasA	-3.111
PA3724	*lasB*	elastase LasB	-3.577
**Genes related to flagellum**			
PA1077	*flgB*	flagellar basal-body rod protein FlgB	-4.257
PA1078	*flgC*	flagellar basal body rod protein FlgC	-3.471
PA1092	*fliC*	flagellin type C	-2.840
PA1094	*fliD*	flagellar capping protein FliD	-1.596
PA1097	*fleQ*	transcriptional regulator FleQ	-3.223
PA1460	*motC*	flagellar motor protein MotC	-2.255
PA1461	*motD*	flagellar motor protein MotD	-2.294
PA3350	*flgA*	flagellar basal body P-ring formation protein FlgA	-2.277
**Genes related to type IV fimbriae**			
PA0395	*pilT*	twitching motility protein PilT	-1.945
PA0396	*pilU*	twitching motility protein PilU	-2.196
PA0408	*pilG*	pilus biosynthesis/twitching motility protein PilG	-1.157
PA4525	*pilA*	type 4 fimbrial protein PilA	-3.466
PA4526	*pilB*	type 4 fimbrial biogenesis protein PilB	-2.216
PA4528	*pilD*	type 4 prepilin peptidase PilD	-1.621
**Genes related to exopolysaccharide**			
PA2232	*pslB*	mannose-1-phosphate guanylyltransferase PslB	-1.746
PA2233	*pslC*	glycosyltransferase PslC	-1.743
PA2234	*pslD*	polysaccharide biosynthesis protein PslD	-2.467
PA3385	*amrZ*	alginate and motility regulator Z	-1.316
PA5322	*algC*	phosphomannomutase AlgC	-1.793

**Genes related to adhesin**			
PA4624	*cdrB*	cyclic diguanylate-regulated TPS partner B CdrB	-1.355

Differential gene expression analysis was performed using DESeq2. Genes with |log2 fold change|≥1 and adjusted *P*-value<0.05 were considered significantly differentially expressed.

Table S6. Genome-wide analysis of Gp53 target genes identified by DAP-seq.

Gene ID	Gene name	Description	Fold Enrichment
PA2567	PA2567	hypothetical protein	104.30
PA1913	PA1913	hypothetical protein	98.77
PA3722	PA3722	hypothetical protein	96.60
PA1727	*mucR*	bifunctional diguanylate cyclase/phosphodiesterase	95.16
PA4843	*gcbA*	diguanylate cyclase GcbA	78.52
PA3340	PA3340	hypothetical protein	77.50
PA3385	*amrZ*	alginate and motility regulator AmrZ	72.10
PA2463	PA2463	hypothetical protein	63.05
PA3762	PA3762	hypothetical protein	55.27
PA1131	PA1131	probable major facilitator superfamily (MFS) transporter	46.92
PA4844	*ctpL*	CtpL	44.21
PA3353	*flgZ*	flagellar motility	42.96
PA3740	PA3740	hypothetical protein	40.46
PA3662	PA3662	hypothetical protein	31.88
PA3540	*algD*	GDP-mannose 6-dehydrogenase AlgD	21.45
PA2867	PA2867	hypothetical protein	19.50
PA4520	PA4520	probable chemotaxis transducer	12.01
PA0807	*ampDh3*	AmpDh3	10.76
PA3350	*flgA*	flagellar basal body P-ring formation protein FlgA	9.90
PA4033	*mucE*	MucE	9.21
PA0082	*tssA1*	type VI secretion protein	9.03
PA1736	PA1736	probable acyl-CoA thiolase	8.97
PA2231	*pslA*	glycosyltransferase PslA	8.20
PA3343	*hsbD*	diguanylate cyclase HsbD	7.12
PA0472	*fiuI*	FiuI	6.86
PA4812	*fdnG*	formate dehydrogenase-O, major subunit	6.78
PA1851	*imcA*	hypothetical protein	6.77
PA4601	*morA*	bifunctional diguanylate cyclase/phosphodiesterase	6.67
PA0938	*wzz2*	Wzz2	5.96
PA1899	*phzA2*	phenazine biosynthesis protein PhzA2	5.91
PA1874	PA1874	hypothetical protein	5.83
PA4683	PA4683	hypothetical protein	5.61

PA2883	PA2883	hypothetical protein	5.55
PA3110	PA3110	hypothetical protein	5.26
PA4952	PA4952	conserved hypothetical protein	4.73
PA0102	*psCA1*	beta-carbonic anhydrase	4.70
PA1939	PA1939	hypothetical protein	4.61
PA3141	*wbpM*	nucleotide sugar epimerase/dehydratase WbpM	4.61
PA2426	*pvdS*	sigma factor PvdS	4.60
PA0180	*cttP*	chemotactic transducer for trichloroethylene positive chemotaxis, CttP	4.59
PA5380	*gbdR*	GbdR	4.40
PA1097	*fleQ*	transcriptional regulator FleQ	3.99
PA2204	PA2204	putative binding protein component of ABC transporter	3.78
PA3713	*spdH*	spermidine dehydrogenase, SpdH	3.67
PA1656	*hsiA2*	type VI secretion protein	3.51
PA1646	PA1646	probable chemotaxis transducer	3.13
PA0094	*eagT6*	EagT6	3.03
PA0172	*siaA*	SiaA	2.96
PA2939	PA2939	probable aminopeptidase	2.93
PA2282	PA2282	hypothetical protein	2.93
PA0674	*vreA*	VreA	2.84
PA0042	PA0042	hypothetical protein	2.73
PA4349	PA4349	hypothetical protein	2.64
PA2820	PA2820	hypothetical protein	2.60
PA0179	PA0179	probable two-component response regulator	2.47
PA3064	*pelA*	pellicle/biofilm biosynthesis protein PelA	2.42
PA4969	*cpdA*	cyclic AMP (cAMP) Phosphodiesterase, CpdA	2.30
PA1107	*roeA*	diguanylate cyclase	2.28
PA3177	PA3177	hypothetical protein	2.04
PA5157	PA5157	probable transcriptional regulator	2.00

Table S7. Predicted amino acids of Gp53 involved in DNA binding based on molecular docking.

Motif	Amino acids
Gp53-m1-1	Thr6, Arg7, Arg15, Arg21, Ser34, Met35, Asn36
Gp53-m1-2	Phe4, Thr6, Arg7, Arg15, Arg21, Ser34, Met35, Asn36
