# Supplementary material for: The bacteriophage transcription factor Gp53 suppresses cell division and regulates Pseudomonas aeruginosa physiology
Source: Commun Biol. 2026 Oct 8;9:1275. doi: 10.1038/s42003-026-11031-z (PMC13650114; doi:10.1038/s42003-026-11031-z)
Supplement: Supplementary file 2 [file 42003_2026_11031_MOESM2_ESM.pdf]

Description of Additional Supplementary Files

Supplementary Data 1: Source data for the predicted Gp53 tetramer model.

Supplementary Data 2: The numerical source data underlying the main figures.
